# A bimodal type of AgPd Plasmonic Blackbody Nanozyme with boosted catalytic efficacy and synergized photothermal therapy for efficacious tumor treatment in the second biological window

**DOI:** 10.1186/s12951-022-01627-y

**Published:** 2022-09-24

**Authors:** Tao Jia, Dan Li, Jiarui Du, Xikui Fang, Valeriy Gerasimov, Hans Ågren, Guanying Chen

**Affiliations:** 1 MIIT Key Laboratory of Critical Materials Technology for New Energy Conversion and Storage, School of Chemistry and Chemical Engineering & State Key Laboratory of Urban Water Resource and Environment & Key Laboratory of Micro-systems and Micro-tructures Ministry of Education, Harbin Institute of Technology, Harbin, 150001 People’s Republic of China; 2 International Research Center of Spectroscopy and Quantum Chemistry, Siberian Federal University, Krasnoyarsk, 660041 Russia; 3 Institute of Computational Modelling, Federal Research Center KSC SB RAS, Krasnoyarsk, 660036 Russia

**Keywords:** Nanozyme, Tumor microenvironment, Theranostics, Plasmonic, Black body

## Abstract

Nanozymes are promising for precise cancer treatment, but are typically limited in terms of the low catalytic efficiency and the complexity in tumor microenvironment (TME). Herein, we describe a bimodal type of AgPd plasmonic blackbody (AgPd PB) nanozyme of compact sizes (< 30 nm), which presents not only boosted enzyme efficacy but also efficient photothermal therapy (PTT) for synergized therapy through tissue-penetrating light in the second biological window (1000–1700 nm). The synthesized hyperbranched AgPd PB nanozymes possess intense and broadband localized surface plasmonic resonance absorption of 400–1300 nm, entailing prominent photothermal efficiency (*η* = 45.1% at 1064 nm) for PTT. Importantly, PTT was found to significantly boost the nanozyme efficacy of both catalase (CAT) and peroxidase (POD) processes, which correspondingly decompose H_2_O_2_ to into O_2_ to relieve tumor hypoxia, and activate H_2_O_2_ to generate oxidative •OH radical. While the generated •OH was found to be able to minimize heat shock proteins (HSPs), which plays a vital role to counterbalance PTT effect both in vitro and in vivo. As compared to control ground without treatment, the synergized nanozyme and PTT activities resulted in about 7-fold reduction of tumor volume, thus elevating the survival rate from 0 to 80% at 30 days posttreatment. Besides the synergistic therapy, the AgPd PB nanozyme were shown to own fluorescence, computed tomography (CT), and photoacoustic (PA) imaging abilities, thus having implications for uses in imaging-guided precise cancer therapy. This study provides a paradigm of TME responsive theranostics under NIR-II light irradiation.

**Graphical Abstract:**

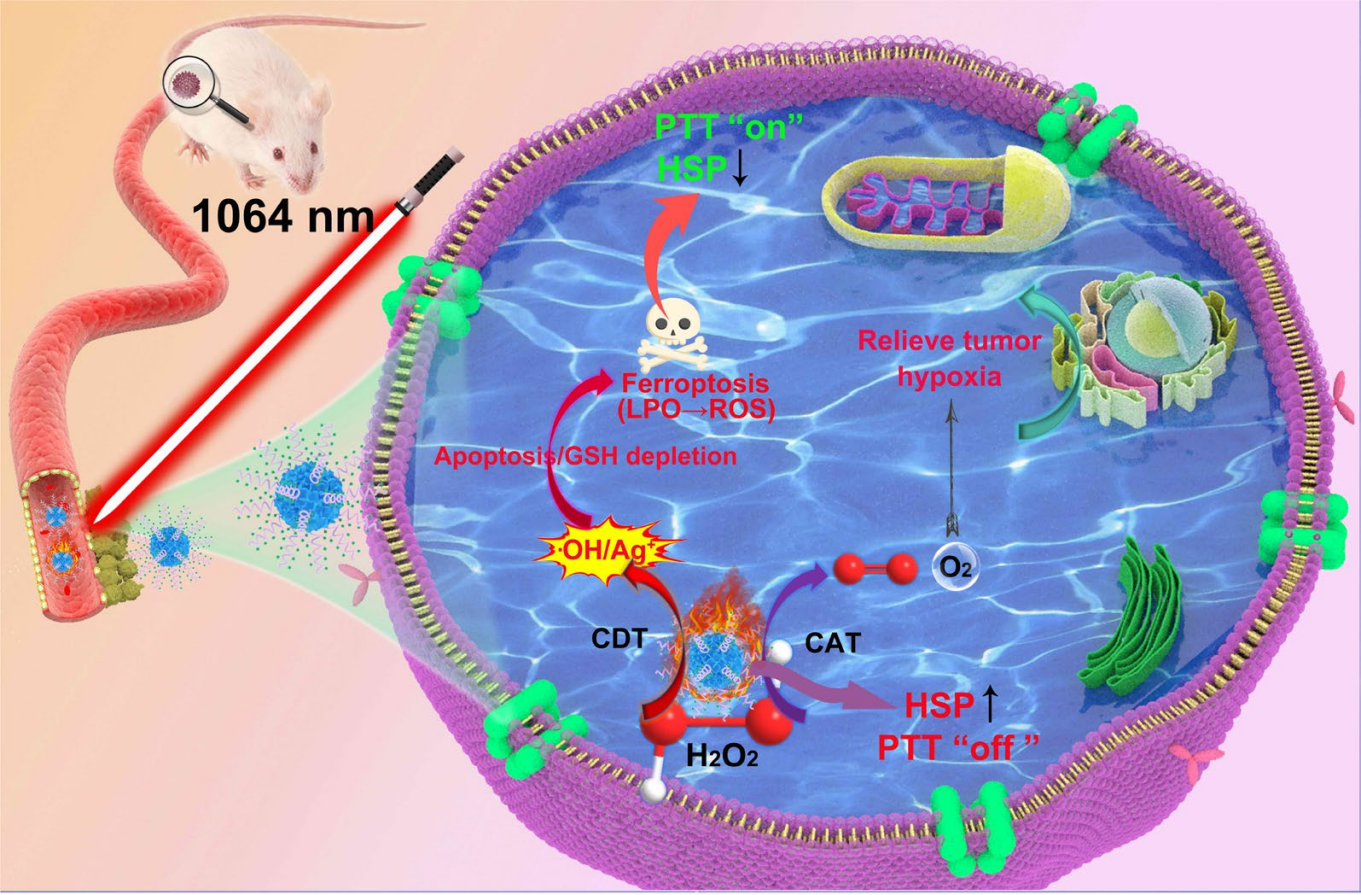

**Supplementary Information:**

The online version contains supplementary material available at 10.1186/s12951-022-01627-y.

## Introduction

Nanozymes hold promise to complement natural protein enzymes to regulate biological processes, as they have superior advantages such as stable structure, enriched catalytic sites, abundant enzymatic activities, and robust catalytic activities under even harsh conditions (acid, base, and high temperature) [1–10]. Indeed, these advantages make nanozymes promising as nanotheranostic agents for applications ranging from biomedical detection to disease treatment, especially in tumor microenvironment (TME) responsive theranostics [11–15]. TME is characterized by mildly acid conditions (pH 6-6.5), hypoxia (1% of O_2_), overproduction of H_2_O_2_ (50–100 × 10^− 6^ M) and glutathione (GSH) (≈ 10 × 10^− 3^ M) [16–20]. Tumor hypoxia weakens the therapeutic effects by photodynamic therapy, sonodynamic therapy and radiotherapy, and plays a crucial role in tumor metastasis and proliferation. While overexpressed H_2_O_2_ and GSH counterbalance the outcome of standard chemotherapy and radiation therapy, by disrupting the regular cell pathways responding to oxidative stress. On the other hand, this also provides opportunities to alleviate hypoxia and produce reactive oxygen species (ROS), such as hydroxyl radicals (•OH), for effective tumor treatment through nanocatalytic activities—chemodynamic therapy (CDT) using enriched H_2_O_2_ [21–25]. A great deal of effort has been taken to explore the utilization of nanozyme for catalytic tumor therapy [26–34], which, however, was confined by the low catalytic efficiencies.

Enzyme activities have long known to be impacted by the local temperature of the enzyme [35–39]. An *in-situ* regulation of enzyme eigen temperature is able to regulate the catalytic efficiencies in vitro and in vivo, favoring tumor treatment outcome. Photothermal effect is a phenomenon associated with material absorption of electromagnetic waves, resulting in the production of thermal energy (heat). This effect has been utilized for effective cancer treatment, i.e., photothermal therapy (PTT), by localized non-invasive therapeutic interventions through tumor-seated photothermal agents, which generate hyperthermia to kill tumor cells (*via* heating them to 42–45 °C or even higher) [40–49]. Indeed, marriage of PTT with nanozyme (using Fe_3_O_4_ nanozyme and Pd nanosheet complexes, and platinum nanozymes coated with porous gold nanoshell) was observed to yield an enhanced tumor theranostics through visible or near infrared light in the first biological window (NIR-I, 650–950 nm) [50–54]. However, the synthesis procedures of these hybrid materials are complicated and stringent. Moreover, realization of noninvasive remote control of nanozyme activities in living systems is challenging, as the utilized visible and NIR-I light has limited tissue penetration depth due to the existence of substantial light scattering in biological tissues. Photon scattering scales as λ^−α^, where lambda is the wavelength and α = 0.2 ‒ 4 for biological tissues [55]. As a result, light in the NIR-II region (1000–1750 nm) can reach maximal centimeter-depth in biological tissues as evidenced from recent imaging results [56–63], promising the use of NIR-II light for remote photocontrol of enzymatic efficiencies.

**Scheme 1:**
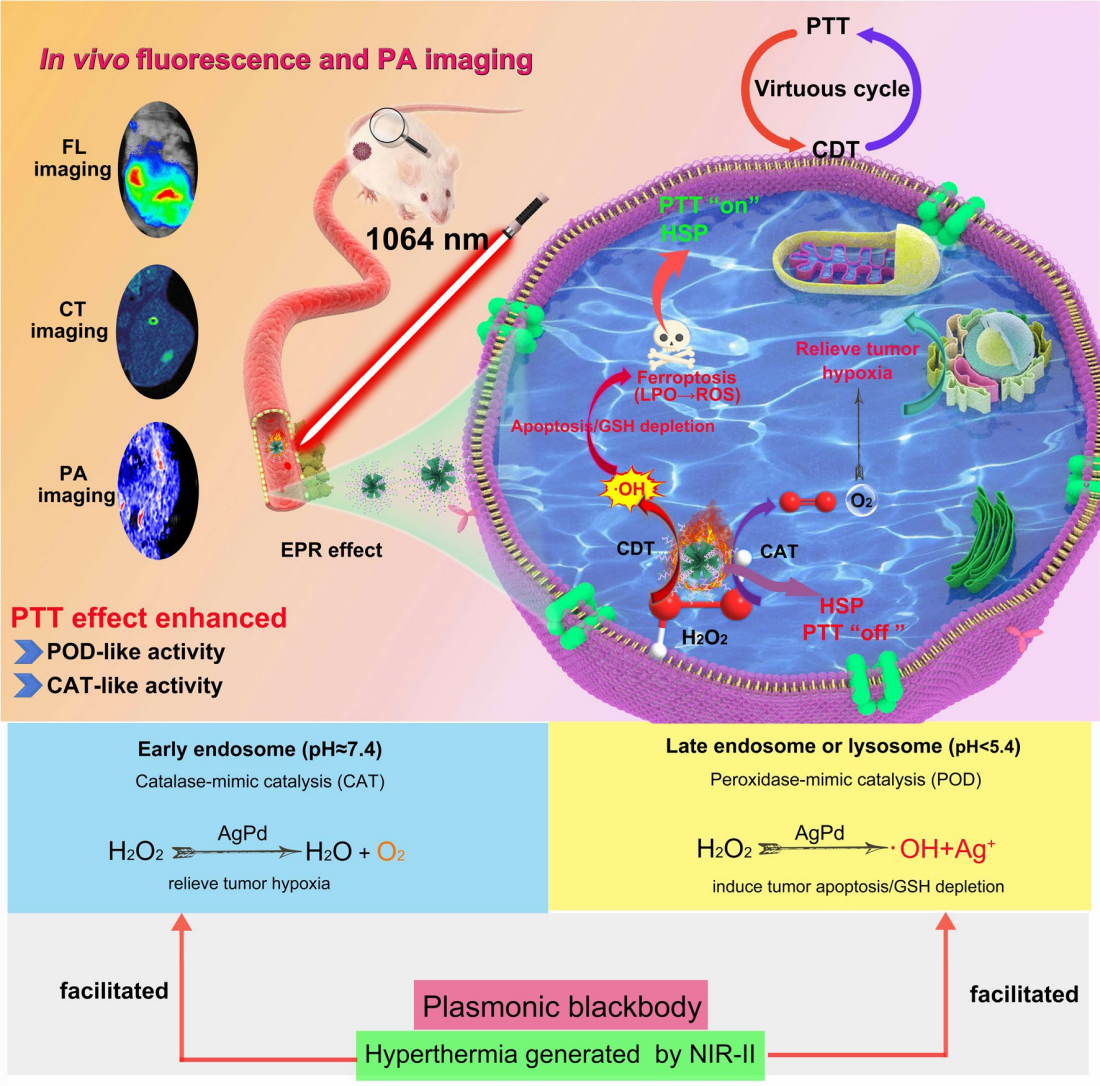
Schematic illustration of the transport of AgPd PB nanozyme in blood vessel, EPR-mediated tumor accumulation, combined PDT/CDT mechanism, and multiple imaging abilities

Herein, we describe a bimodal type of hyperbranched AgPd plasmonic blackbody (AgPd PB) nanozyme of compact size (< 30 nm), which not only exhibits nanozyme activities but also can be in situ heated in vivo through tissue-penetrating NIR-II light. These AgPd PB nanoparticles have intense localized surface plasmonic resonance (LSPR) absorption in a broad spectral range across 400–1300 nm (molar extinction coefficient 2.0 × 10^9^ mol^− 1^·cm^− 1^ at 1064 nm), delivering prominent photothermal efficiency (*η* = 45.1%). Indeed, we verified that the PTT can significantly elevate the catalyze-mimic (CAT) activity to relieve tumor hypoxia, as well as the peroxidase-mimic (POD) activity, which produces toxic hydroxyl radicals (•OH) that can induce the apoptosis of tumor cells. Moreover, the generated •OH radical can suppress the expression of heat shock protein (HSPs) that triggers tumor cell self-preservation, thus maximizing the PTT treatment results. The synergized nanozyme and PTT enable an efficacious antitumor therapy both in vitro and in vivo. (Scheme 1).

## Experimental section

### Chemicals and characterizations

Chemicals and Reagents. Sodium borohydride (NaBH_4_), cetyltrimethylammonium chloride (CTAC), palladium chloride acid (H_2_PdCl_4_) and silver nitrate (AgNO_3_) were purchased from Sigma-Aldrich, 3,3′,5,5′-tetramethyl-benzidine (TMB), Ascorbic acid (AA), 2,7-dichlorofluorescein diacetate (DCFH-DA), MTT, 5,5-dimethyl-1pyrroline N-oxide (DMPO), 4’,6-diamidino-2-phenylindole (DAPI), fluorescein isothiocyanate (FITC), SH-PEG_5000_-NH_2_, 7-hydroxycoumarin, were received from Aladdin (Shanghai, China). JC-1 staining kit was purchased from Beyotime Inst. Biotech. (Haimen, China). The Hela cell line and U14 cell line were purchased from FDCC (Ruilu in Shanghai, China). All of the chemical reagents involved in this article were used without further purification.

TEM images were obtained from a FEI Tecnai G2 S-twin transmission electron microscope. XRD was measured by Japanese Neo-Japanese TTR-III diffractometer. ICP measurements were gained by ICP-5000 concentrating plasma emission spectrometer. FTIR was obtained by the Thermo Scientific NicoleTiS 5 Fourier transform infrared spectrometer. Leica SP8 was utilized to gain Confocal laser scanning microscopy (CLSM) images. Mice were subjected to gas anaesthesia experiment by MATRXVMR small animal gas anaesthesia machine. In vivo imaging of mice was measured by Fluorvivo in vivo imaging instrument. CT imaging of mice was measured by MicroCT of PerkinElmer Quantum GX type. The cell apoptosis was detected by flow cytometer (CytoFLEX, Beckman).

### Synthesis of branched Ag33%Pd67% PB nanozyme

A simple seed-mediated growth method was applied. In brief, cold NaBH_4_ (0.01 M, 600 µL) was added to a mixture of the CTAC (0.1 M, 9.75 mL) and H_2_PdCl_4_ (0.01 M, 250 mL) to prepare a seed solution. After gently stirring, the seed solution remained undisturbed for 2 h at room temperature. The growth solution was a mixture of CTAC (4.5 mM, 10 mL), H_2_PdCl_4_ (0.01 M, 0.3 mL) and AgNO_3_ (0.01 M, 0.3 mL) with a pH value of 4. After a quick injection of seed solution (40 µL) into the growing solution, the freshly prepared ascorbic acid (AA, 0.1 M, 100 mL) is then added rapidly. The synthetic solution was kept at room temperature for 30 min. The branched AgPd nanozyme product were obtained after centrifugation (7000 rpm) for twice and then dispersed in deionized water. Then, the AgPd product solutions were then added with 20 mg SH-PEG-NH_2_ and kept under a continuous stirring about 6 h at -4 ℃. After centrifugations and washed by ultrapure water for multiple times (7000 rpm), the final product AgPd-PEG-NH_2_ was gained.

### In vitro O_2_ production

AgPd PB nanozyme (100 µg·mL^− 1^) was mixed with H_2_O_2_ solution (50 µM), and the O_2_ production at diverse time intervals (0, 20, 40 and 60 min) were measured by O_2_ meter instrument (JPBJ-608).

### In vitro cell phagocytosis experiment

HeLa cells were cultivated overnight in a 6-well plate to obtain monolayer cells (7000–8000 per well). Later fluorescence dye FITC-conjugated AgPd PB nanozyme (100 µg·mL^-1^) was added into the each well and incubated further for 0.5, 1, 1.5 and 2 h. Afterwards, the cells were washed with PBS for three times to remove additional materials, and stained with DAPI (20 ug·mL^-1^, 10 min) for CLSM fluorescence imaging of cells.

### In vitro cytotoxicity and biocompatibility assays

A typical MTT assay was applied to evaluate the in vitro cytotoxicity of prepared samples. Typically, HeLa cells were seeded in a 96-well plate for 12 h to obtain monolayer cells, then samples AgPd PB nanozyme of various concentrations (6, 12, 25, 50, 100 and 200 µg·mL^-1^) were added into cell culture medium for another 3 h culture to allow efficient cell uptake. Later the HeLa cells were dived into five groups with the following conditions: Control (without treatment), laser irradiation (NIR), AgPd PB nanozyme, AgPd PB nanozyme with AA plus laser (AgPd PB + AA + NIR), and AgPd PB nanozyme plus laser irradiation (AgPd PB + NIR) with laser (1.25 W·cm^-2^) irradiation time was about 5 min. Afterwards the culture medium was removed, then 20 µL of MTT solution (5 mg·mL^-1^) was added to each well for 4 h incubation. Afterwards, 130 µL of DMSO was added to each well and the absorbance at 490 nm was detected. Similar MTT assay was also utilized to evaluate the in vitro viability effect of L929 fibroblast cells, induced by added different concentrations of AgPd PB nanozyme (31, 62, 125, 250 and 500 µg·mL^-1^). Apoptosis was analyzed by Annexin V-FITC and PI method. Totally, HeLa cells were seeded in 6-well plates at 8000 − 1000 cell well^-1^ density and incubated for 24 h. Afterwards, a new acidified DMEM containing H_2_O_2_ (100 µM) was added for another 4 h of culture. Cells treated similarly with MTT assays, after an additional 20 h with AgPd PB nanozyme (100 µg·mL^-1^), cells were washed 3 times with PBS and treated with Annexin V-FITC (5 uL) and PI (5 uL). Cell apoptosis was quantified by flow cytometry.

### ESR measurements

For the detection of •OH, solution of AgPd PB nanozyme (100 µg·mL^-1^) and spin traps (traps DMPO for •OH) (10 mM) mixing solution was added H_2_O_2_ (50 µM). After a gentle shake for 5 min, about 100 µL solution was transferred to a quartz capillary tube for further analysis. ESR spectra were measured using a Bruker EMX EPR spectrometer.

### Hemolysis test

Hemolysis test was conducted using human blood. Firstly, 2% red blood cell suspension was centrifugally washed with 0.9% sodium chloride isotonic solution for multiple times, and then the blood samples were diluted with phosphate saline. 0.5 mL of cell suspension and 2 mL of hydrochloric acid buffer were mixed as positive control, while 2 mL of hydrochloric acid buffer was used as negative control. 2 mL samples (12.5, 50, 100, 200, 400 and 800 µg·mL^-1^) and a mixture of hydrochloric acid buffer were left standing at room temperature for 6 h. The supernatants were obtained by centrifugation at 10,000 rpm for 5 min, and the absorbances at 541 nm of samples were measured by UV–vis spectrometer. The hemolysis rate was calculated by the blew formula:

$${\text{Hemolysis}}\,{\text{ratio}}\, = \left( {{\text{A}}_{{{\text{samples}}}} - {\text{A}}_{{{\text{negative}}\,{\text{control}}}} } \right)/\left( {{\text{A}}_{{{\text{positive}}\,{\text{group}}}} - {\text{A}}_{{{\text{negative}}\,{\text{control}}}} } \right)$$

### Detection of intracellular ROS

DCFH-DA kit was used to detect the ROS generation. HeLa cells were cultured in the 6-well plate for 12 h to gain the monolayer cells, then 1 mL PBS solution of AgPd PB nanozyme (100 µg·mL^-1^) was mixed for another 3 h of cultivation. Afterwards, DCFH-DA was added and cultured for another 0.5 h. After 5 min irradiation of 1064 nm laser, the fluorescence images of ROS were gained by CLSM.

### Detection of intracellular O_2_ production

HeLa cells were cultured with Ru(dpp_3_)Cl_2_ (50 µM) for 4 h, later 1 mL of AgPd PB nanozyme solution (100 µg·mL^-1^) was added and incubated for diverse time intervals (20, 40 and 60 min). After washed by PBS for several times, 1 mL of DAPI was added to dye the cell nucleus for 10 min. Finally, the fluorescence photos were observed by CLSM.

### Mitochondrial integrity assay

HeLa cells were incubated into six-plates to obtain adherent cells. Then AgPd PB nanozyme (150 µg·mL^-1^) was added for 4 h of culture. Then the cells were treated with diverse conditions: Control (without treatment), laser irradiation (NIR), AgPd PB nanozyme (AgPd PB), AgPd PB nanozyme with AA (AgPd PB + AA) and AgPd PB nanozyme plus laser irradiation (AgPd PB + NIR). After another 2 h of culture, JC-1 (15 µg·mL^-1^) solution was added for 20 min. Later HeLa cells were cleaned with PBS and dyed with DAPI for another 10 min. Afterwards, the fluorescence images of cells were accumulated by CLSM.

### Detection of intracellular •OH production

HeLa cells were seeded overnight in the 6-plate, then AgPd PB nanozyme (100 µg·mL^-1^) was added for another 2 h of culture. Next 1 mL of 7-hydroxycoumarin (5 × 10^− 4^ M) was mixed for 1 h in a dark room. After irradiation for diverse time (20, 40 and 60 min) by 1064 nm laser, the intracellular fluorescence was recorded by CLSM.

### In vivo and in vitro X-ray CT imaging

The AgPd PB nanozyme was dispersed in phosphate buffers at diverse concentrations of 1.25, 2.5, 5, 10 and 20 mg·mL^-1^, subsequently they were added to a 1.5 mL test tube for in vitro CT imaging. In order to perform in vivo CT imaging, gas anesthesia was first performed using a small animal anesthetic machine. Afterwards, 200 µL of AgPd PB nanozyme (20 mg·mL^-1^) was injected in situ into tumor-bearing mice. The CT scans were obtained on the mice before and after the intratumorally injection of the reagent.

### In vivo photothermal imaging

The tumor-bearing mice at 12 h post-injection of AgPd PB nanozyme with saline were irradiated with 1060 nm laser irradiation for 5 min. Meanwhile, the IR thermal images of temperature at tumor sites were recorded with an IR camera.

### In vivo photoacoustic imaging and blood oxygen imaging

Tumor bearing mice were anesthetized with gas and intravenously injected with AgPd PB nanozyme (20 mg·mL^-1^). The corresponding photoacoustic imaging of diverse times points (0, 3, 6 and 8 h) was collected for further analysis. For the blood oxygen imaging, the anesthetized mice were intratumorally injected with AgPd PB nanozyme (20 mg·mL^-1^) and irradiated with 1064 nm laser for 30 and 60 s. The synchronous imaging was recorded for analysis.

### In vivo fluorescence imaging

The biodistribution of AgPd PB nanozyme were gained by utilizing the in vivo imaging system (In Vivo FPRO). Tumor-bearing mice were anesthetized and tested the in vivo fluorescence after intravenous injection of AgPd PB nanozyme conjugated with Rhodamine B (10 mg·mL^-1^, 150 µL). Subsequently, the mice were dissected immediately to obtain heart, liver, spleen, lung, kidney and tumor for fluorescence examination. Afterwards, the relative organs and tumors were dissolved in concentrated nitric acid solution, and the Ag and Pd ions were quantified by ICP-OES after proper dilution of water.

### Western blot analysis

The expression levels of HSP 70 in HeLa cells were detected. HeLa cells were seeded in 6-well plates (8000–10,000 per well). HSP 70 detection was divided into four groups: (1) Control group; (2) NIR irradiation (1.2 W·cm^-2^, 5 min); (3) AgPd PB (100 µg·mL^-1^); (4) AgPd PB + NIR + AA. (5) AgPd PB + NIR. The cells were then collected with trypsin and lysed. Subsequently, the protein content was detected by BCA protein assay. The proteins are separated with sodium dodecyl ammonium sulfate-polyacrylamide gel electrophoresis (SDS-PAGE) and then with polyvinylidene fluoride (PVDF) membrane. After blocking with 5% dry skim milk for 1 h and spending the night on a shaker with the corresponding primary antibody (4 ºC) and cultured with secondary antibody (room temperature, 1 h), the membrane was visualized using the ECL plus detection system.

### In vivo toxicity and histological tests

Female Kunming mice with an average weight of approximate 20 g were supplied by the Experimental Animal Center of Harbin Medical University. All animal experiments were approved by the Ethics Committee of the Second Affiliated Hospital of Harbin Medical University (Harbin, China). Animal experimental procedures were executed in accordance with the Guidelines for Care and Use of Laboratory Animals of the Drug Safety Evaluation Center of Harbin Medical University (No. SYDW 2019-82). U14 tumor cells were injected subcutaneously into the right hind leg and cultured for 7 days to observe the survival of tumor-bearing mice. Tumor bearing-mice were divided randomly into five groups with (5 mice/group. They were (1) PBS treated group (control), 1064 nm irradiation group (NIR), AgPd PB nanozyme, AgPd PB nanozyme + NIR + AA and AgPd PB nanozyme + NIR. A 1064 nm near-infrared laser is irradiated at a power density of about 1.25 mW·cm^-2^ for 5 min. Tumor volume and body weight of each mouse were assessed daily after treatment. Two weeks after the first treatment of tumor-bearing mice, all mice were anesthetized and euthanized. Tumor and major organs (liver, lung, heart, kidney and spleen) were taken for photographing and hematoxylin & eosin (H&E) and TUNEL staining to analyze the histological and pathological changes.

## Results and discussion

### Synthesis and Characterization of AgPd PB nanozyme

**Fig. 1:**
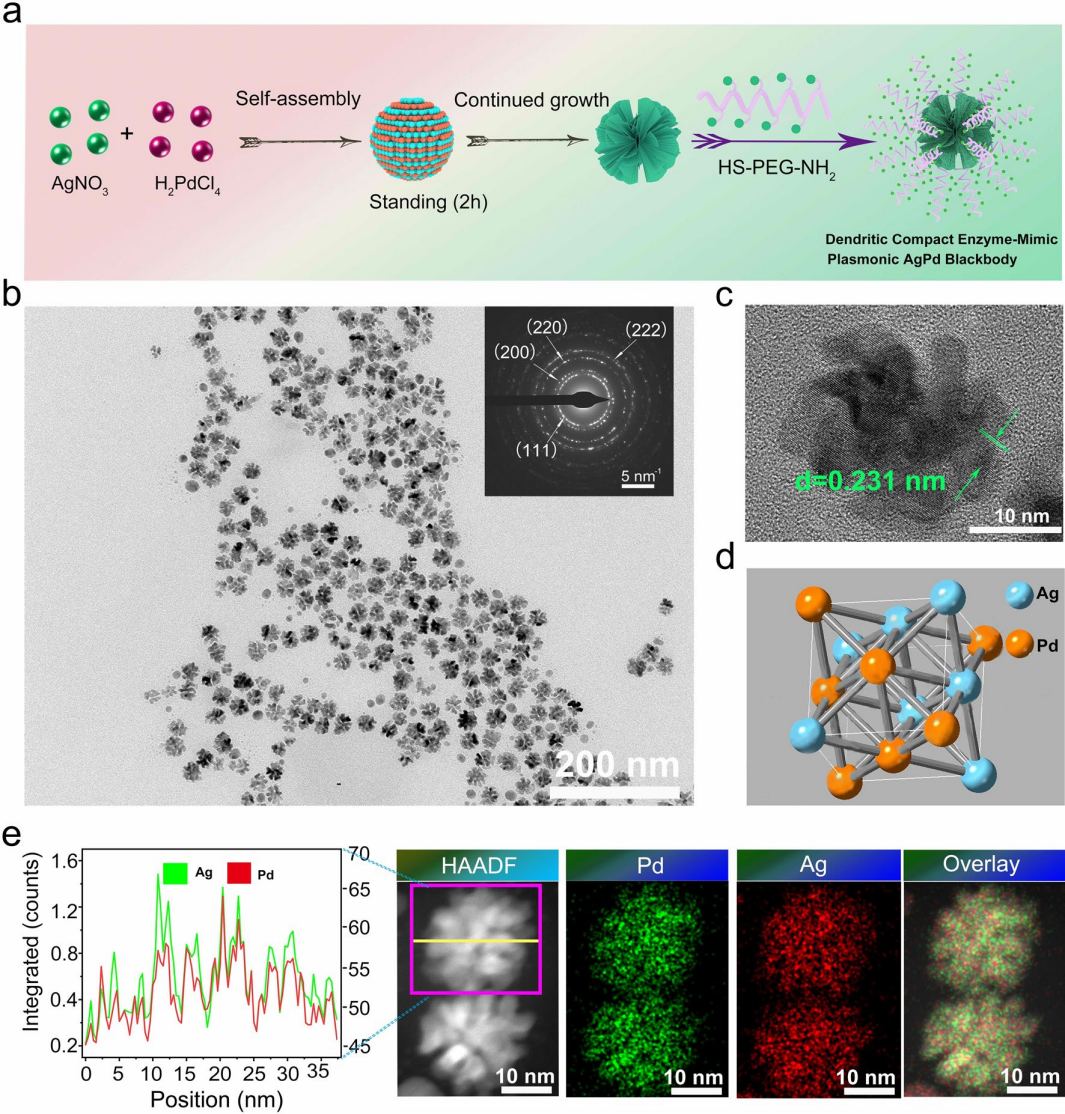
**a** Schematic illustration for the synthesis of AgPd PB nanozyme. **b** TEM and **c** high resolution TEM of AgPd PB nanozyme, with **d** crystal structure shown in (Pd and Ag atoms are colored in orange and blue, respectively). **e** Elemental mapping of Pd and Ag (right) in a single AgPd nanostructure shown in the HADDF-STEM image, as well as the content distribution along the line (highlight in yellow) across the single AgPd PB nanozyme (left)

The synthesis procedure of AgPd PB nanozyme was outlined in Fig. 1a. Briefly, H_2_PdCl_4_ solution and cetyltrimethylammonium chloride (CTAC) were mixed firstly, after which fresh sodium borohydride (NaBH_4_) was added as reducer to obtain Pd seeds solution. Subsequently, H_2_PdCl_4_, AgNO_3_, and fresh ascorbic acid (AA) were further added to the Pd seeds solution, of which A 2 h standing can yield nanoflower-like AgPd PB nanozymes. A ligand SH-PEG-NH_2_ was further coated onto AgPd PB to accomplish the final product AgPd-PEG-NH_2_ for further uses. The success of the ligand coating was indicated by the measured zeta potential, which was decreased from 23 mV to 12.4 mV after PEG modification (Additional file 1: Fig. S1a). Furthermore, the stability of the PEGlyated AgPd PB nanozyme in PBS, DMEM, and serum was evaluated by dynamic light scattering (DLS) measurement. Furthermore, the polydispersity index (PDI) changed from 0.19 to 0.15 after PEG modification, proving the superior dispersity of AgPd PB. No significant change of the hydrodynamic particle size was observed after two weeks, indicating the superior stability of the PEGlyated AgPd PB nanozyme (Additional file 1: Fig. S1b).

The as-prepared nanozymes were observed to be monodispersed and possess flower-like shape with a compact size of ~ 30 nm (Fig. 1b). A selected electron diffraction (ED) diffraction patterns (inset of Fig. 1b) indicated the existence of crystals faces of (111), (220), (200) and (222), consistent with the XRD patterns (Additional file 1: Fig. S2a) of the cubic crystal structures illustrated in Fig. 1d. A representative high resolution transmission electron microscopy (HRTEM) image (Fig. 1c) illustrates a clear lattice fringe with a d spacing of 0.231 nm, right between the d spacing of fcc Ag (0.236 nm) and fcc Pd (0.226 nm) (111) plane. An elemental profiling along the yellow line (left) as well as whole mount elemental imaging of a single AgPd nanostructure (right) shown in the high-angle annular dark-field imaging scanning transmission electron microscopy (HADDF-STEM) image (Fig. 1e), confirming the homogenous blending of Ag and Pd elements in the alloyed AgPd PB nanozyme. The energy dispersive spectrometer (EDS) spectrum shown in Additional file 1: Fig. S2b further verified the composition of AgPd PB nanozyme. The formation of solid solution alloying was further supported by X-ray photonelectron spectroscopy (XPS) and corresponding Ag 3d and Pd 3 g region, in which electron binding energy from Pd (334.23 and 339.52 eV) and from Ag (373.9 and 367.9 eV) elements were observed (Additional file 1: Fig. S2c–e).

### Photothermal properties of AgPd PB nanozyme

**Fig. 2:**
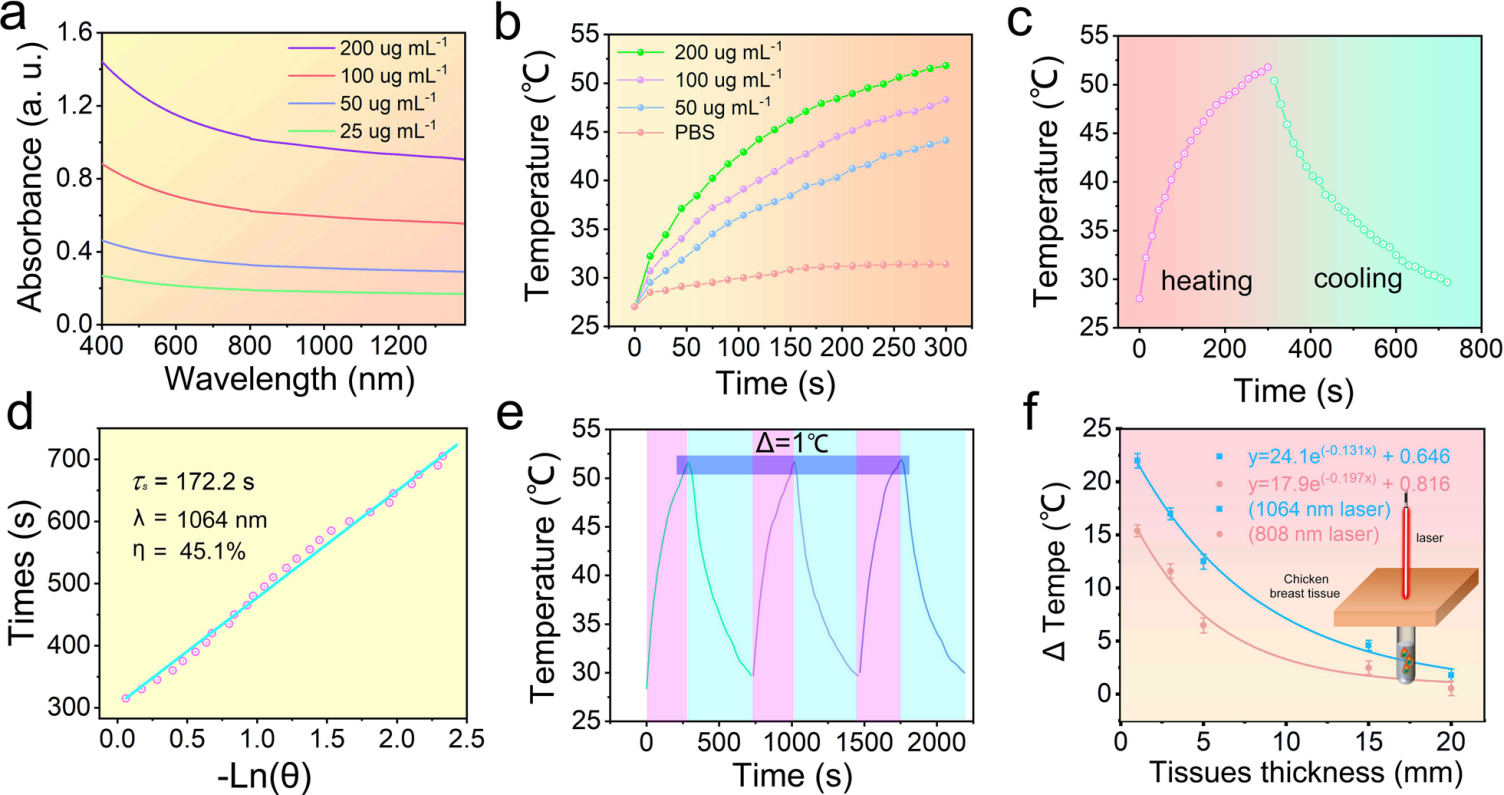
**a** UV-Vis-NIR absorbance spectra of AgPd PB nanozyme of varying concentrations. **b** Temperature change curves of AgPd PB nanozyme aqueous solutions with different concentrations upon 1064 nm laser irradiation. **c** A single heating and cooling cycle of AgPd PB nanozyme under 1064 nm laser irradiation (1.2 W·cm^-2^) and **d** linear time data versus − ln*θ* obtained from the cooling period. **e** Photothermal conversion cycling test of AgPd PB nanozyme under 1064 nm laser irradiation (1.2 W·cm^-2^). **f** Measured temperature change of AgPd PB nanozyme solution (200 µg·mL^-1^) versus the depth of covered chicken breast tissue under 808/1064 nm laser irradiation; an exponential decay function was used to fit the experimental data, while a schematic illustration of the measuring setup was shown in the inset

AgPd PB nanozymes were observed to have broad absorption and efficient photothermal conversion properties. As shown in Fig. 2a, the absorbance spectra of metallic alloyed AgPd PB nanozyme exhibit a wide range of extinction across 400–1300 nm in a concentration-dependent manner. This intense and uniform broadband absorption across is possibly ascribed to the strong intraparticle plasmonic coupling among branches in close proximity. The mass extinction coefficient was evaluated to be 5.01 L g^− 1^·cm^− 1^ at 1064 nm (Additional file 1: Fig. S3b), higher than that of reported Cu_9_S_5_ [64], Cu_2 − x_Se [65], BSA-IrO_2_, Au nano-star and CuFe and FePd NIR photothermal agents [66–70] (refer to Additional file 1: Table S1).

Subsequently, photothermal conversion was investigated by measuring temperature change of AgPd PB nanozyme at varying concentrations under laser irradiation at 1064 nm laser (1.2 W·cm^− 2^) (Fig. 2b). The temperature in AgPd PB nanozyme groups was concentration-dependent, which was elevated from 26 to 52 ℃ (about 26 ℃ increase) at AgPd PB nanozyme concentration of 200 µg·mL^− 1^, in stark contrast to PBS group where only 4.3 ℃ increase was observed. Corresponding infrared thermal images of AgPd PB nanozyme under 1064 nm laser irradiation (Additional file 1: Fig. S3c) and temperature change (ΔT) (Additional file 1: Fig. S3d) indicated a fast temperature increase of 24 ℃ within 5 min (AgPd PB concentration of 200 µg·mL^− 1^), further demonstrating the superior photothermal ability. Indeed, photothermal conversion efficiency was calculated to be 45.1% with 1064 nm laser irradiation (Fig. 2c and d), higher that of typically reported photothermal agents of Au-Cu_9_S_5_ Fe_3_O_4_-CuS, and Cu_3_BiS_3_ MXene (refer to Additional file 1: Table S2). Moreover, the photothermal stability of AgPd PB nanozyme was evaluated through heating and cooling cycle curves (Fig. 2e); negligible temperature change was observed after several cycles indicating the excellent stability for photothermal output.

To test the possible use of AgPd PB nanozyme for phototherapy in deep-tissue, AgPd PB nanozyme solution was covered with chicken breast tissue of different thickness (0–20 mm) (Fig. 2f). Both 1064 nm and 808 nm laser (power density 1.2 W·cm^− 2^) were utilized to penetrate chicken breast tissue to heat the AgPd PB nanozyme solution (200 µg·mL^− 1^) for 5 min. As expected, a decreased temperature change (ΔT) was observed upon thickening the covered tissue. However, ΔT always remained higher for 1064 nm than 808 nm for all tissue thicknesses, suggesting better tissue-penetrating ability for 1064 nm than 808 nm which favors in vivo phototherapy. Moreover, even the thickness of chicken breast reached 5 mm, the temperature changes still reached 13 ℃, higher enough to produce adequate PTT effect in deep-seated tumors.

### The catalase-mimic activity (CAT) of AgPd PB nanozyme

**Fig. 3:**
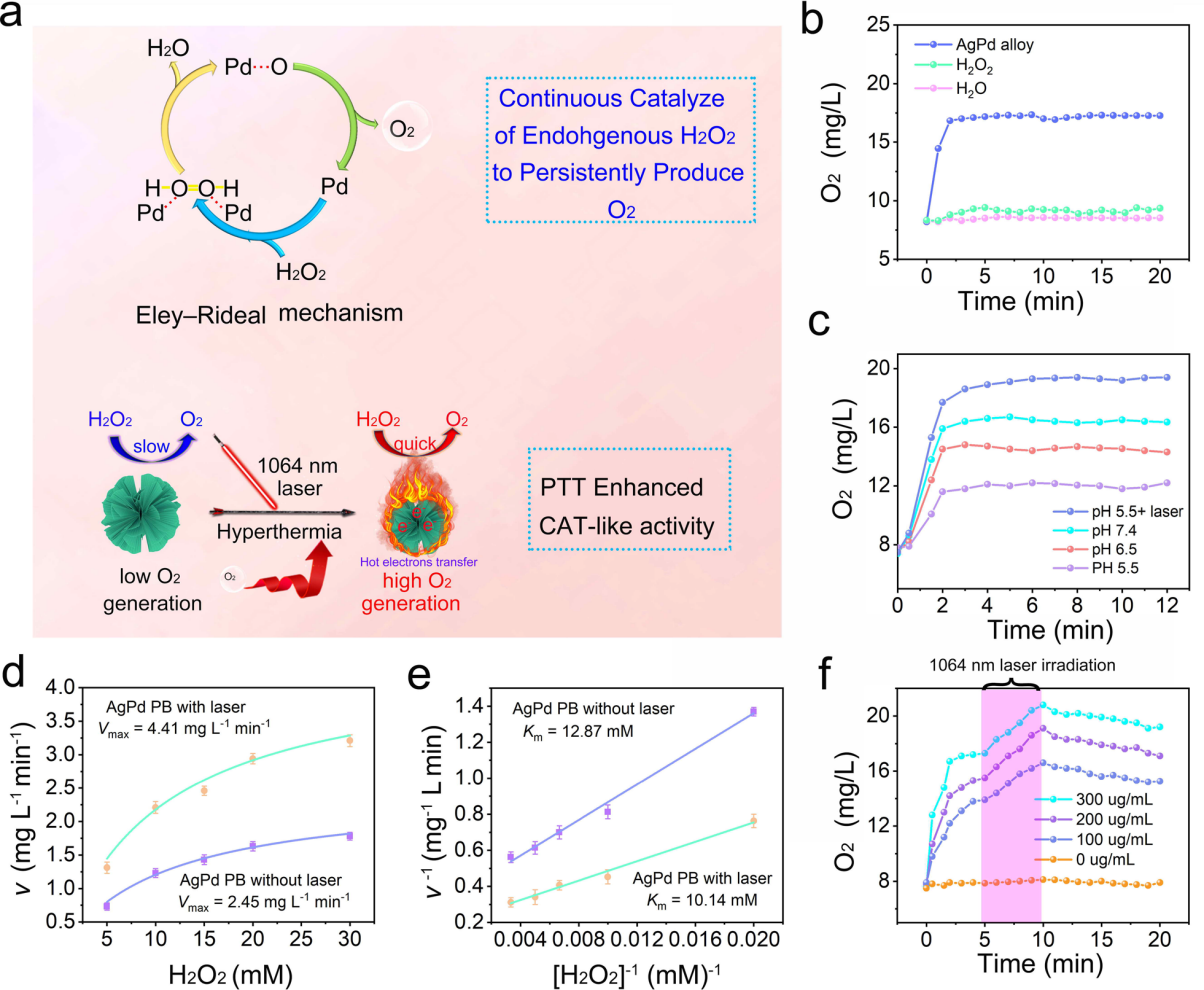
**a** Schematic illustration of AgPd PB nanozymatic processes for producing O_2_ under NIR-II laser irradiation. **b** O_2_ generation test through catalysis of H_2_O_2_ (100 µM in aqueous solution) by AgPd PB nanozyme. **c** O_2_ generation under diverse pH and laser irradiation. **d** Michaelis–Menten kinetic analysis and **e** Lineweaver–Burk plotting for AgPd PB nanozyme with H_2_O_2_ as substrate (pH 5.5). **f** The O_2_ concentration of H_2_O_2_ incubated with different concentration of AgPd PB nanozyme and under irradiation by 1064 nm laser from 5th min to 10th min

Noble metal nanomaterials have been widely investigated in photocatalysis, including water decomposition, H_2_ dissociation, organic synthesis and other related chemical reactions [71–73], yet was rarely investigated for TME-responsive nanozyme activities. We found that AgPd PB nanozyme is able to continuously decompose H_2_O_2_ into O_2_ through Eley–Rideal mechanism. The HO-OH in H_2_O_2_ is first broken to obtain two OH*, which was then formed H_2_O* and O*, and finally converted into H_2_O and O_2_ (Fig. 3a top). Recent studies have demonstrated the capability of plasmonic systems in producing two important effects, the LSPR-induced photothermal effects, and the LSPR-induced hot electrons that can facilitate catalysis when combined with electron-accepting substances [74, 75]. We evaluated the LSPR-enhanced nanozyme effect of AgPd PB by inhibiting PTT through an ice bath, which enabled controlling the temperature of cells less than 37 °C. As shown in Additional file 1: Fig. S4, in comparison with AgPd PB + H_2_O_2_, group of AgPd PB + Ice Bath + H_2_O_2_ + NIR presented an apparent promoting effect in both CAT (a) and POD (b) enzyme activities. By contrast, the laser irradiation group without ice bath exhibited a more significant enhancement, confirming that both the LSPR and the thermal effect could accelerate the catalytic reaction, but the LSPR-induced heating effect on the catalytic process was much higher. Considering the intense LSPR effect of AgPd PB nanozyme, light irradiation triggered hot electrons and generate heat, which can expedite Eley–Rideal cycle, resulting in promotion of the O_2_ generation (Fig. 3a down).

As exhibited in Fig. 3b, the O_2_ concentration from H_2_O_2_ solution rapidly increased from 7.2 mg·mL^− 1^ to 17.6 mg·mL^− 1^ after mixing with AgPd PB nanozyme, while no obvious O_2_ generation was found in pure H_2_O_2_ or H_2_O and AgPd PB mixed solution. We also investigated the catalytic ability of AgPd PB nanozyme under diverse pH conditions. As displayed in Fig. 3c, the catalytic activity of AgPd PB nanozyme was somewhat inhibited with pH descending, but still reached 12.0 mg·mL^− 1^ at pH 5.5 (lower than the typically observed pH values of 6-6.5 in TME). This is possibly due to that the HO-OH is more stable and difficult to break at higher proton concentrations, thus reducing the O_2_ production. Note that after exposure to laser at 1064 nm, the catalytic activity of AgPd PB nanozyme was significantly improved, elevating O_2_ production from 12.0 to18.7 mg·mL^− 1^ (about 1.6-fold increase). Michaelis–Menten kinetic analysis and Lineweaver–Burk plotting were employed to quantity crucial enzymatic parameters of the maximum velocity (*V*_max_) and Michaelis–Menten constant (*K*_m_) using the following equations:

1 $$A = \varepsilon lc$$

2 $${v_0} = \frac{{{V_{\max \cdot}}[S]}}{{{K_m} + [S]}}$$

3 $$\frac{1}{{{v_0}}} = {\rm{ }}\frac{{{K_m}}}{{{V_{\max }}}} \cdot \frac{1}{{[S]}} + \frac{1}{{{V_{\max }}}}$$

The *V*_max_ represents the reaction rate when the enzyme is fully saturated with the substrate, while *K*_m_ reveals the concentration of the substrate when the enzymatic reaction rate reaches half of the maximum, reflecting the affinity between the enzyme and the substrate. As exhibited in Fig. 3d and e, the values of *V*_max_ and *K*_m_ of AgPd PB nanozyme without laser irradiation were calculated to be 2.45 mg·L^− 1^·min^− 1^ and 12.87 mM, while the *V*_max_ and *K*_m_ values of AgPd PB nanozyme with laser irradiation were calculated to be 4.41 mg·L^− 1^·min^− 1^ and 10.14 mM, separately. The increased *V*_max_ and decreased *K*_m_ values demonstrate the apparent enhancement of the catalytic ability of AgPd PB nanozyme due to the expsoure to 1064 nm light. Moreover, the O_2_ production are depdent on AgPd PB nanozyme concentration, with higher O_2_ production at elevated AgPd PB nanozyme concentrations (Fig. 3f and Additional file 1: Fig. S5a). Moreover, for all concentrations of AgPd PB nanozyme, an exposure to 1064 nm laser irradiation between 5 th to 10 th min (Fig. 3f) were observed to accelate O_2_ generation rate, again verifying the promotation of catalytic processs via NIR-II laser induced hyperthermia and LSPR effect. We also compared the capacity of AgPd PB nanozyme with that of typically reported MnO_2_ for O_2_ production. A continous O_2_ production was observed in AgPd PB nanozyme upon repeated addition of H_2_O_2_ aliquots, while inhibition of O_2_ was observed in MnO_2_ at the second and third addition of H_2_O_2_, indicatve of better catalytic endurance capacity of AgPd PB nanozyme than MnO_2_ (Additional file 1: Fig. S5b). Besides, we compared AgPd PB nanozyme with the natural HRP catalase, finding much higher relative catalytic activties than HRP catalse for all investigated high tremperature and hard acid (pH 4–5) conditions (Additional file 1: Fig. S5c).

### The peroxidase-mimic activity (POD) of AgPd PB nanozyme

**Fig. 4:**
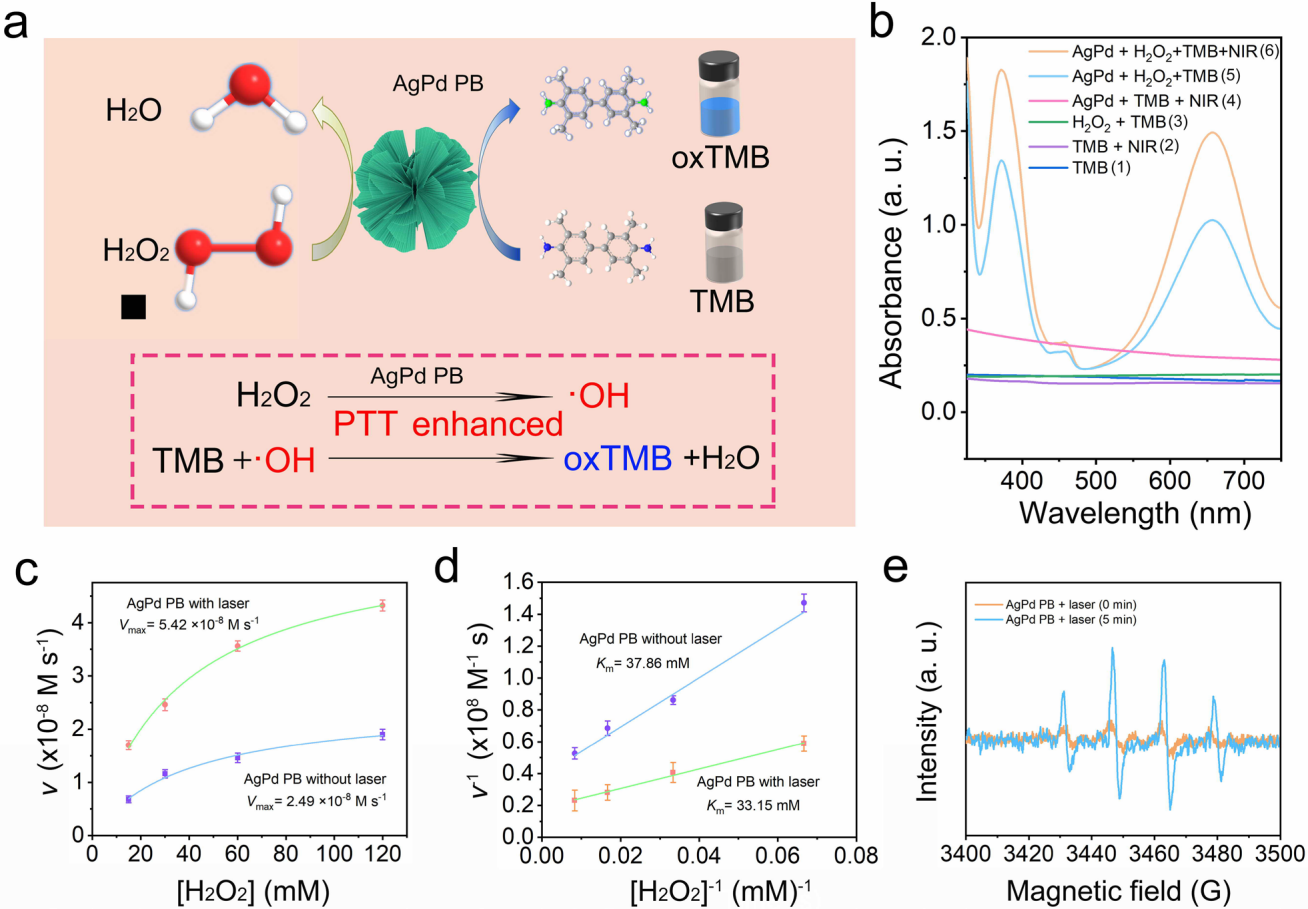
**a** Schematic illustration of the chromogenic reaction ofTMB with AgPd PB nanozyme. **b** UV–vis absorption spectra of the catalyzed oxidation of TMB (oxTMB), as catalyzed by (1) TMB, (2) TMB + NIR, (3) TMB + H_2_O_2_, (4) AgPd + TMB, (5) AgPd + H_2_O_2_ + TMB and (6) AgPd + H_2_O_2_ + TMB + NIR in an acid environment (pH 5.5) (5). Panels at the bottom show the corresponding digital photos of each group. The corresponding **c** Michaelis–Menten kinetics and **d** Lineweaver–Burk plotting upon the addition of varied concentrations of H_2_O_2_ (120, 60, 30 and 15 mM) without and with laser irradiation. **e** ESR spectra of different reaction systems with the DMPO as the spin trap

Along LSPR effect, Ag and Pd was reported to be capable of reacting with H_2_O_2_ under acidic conditions to produce •OH, mimicking POD activities. To probe the ability of alloyed AgPd PB nanozyme to produce •OH *via* Fenton-like reaction, chromogenic detection of •OH was performed using 3,3’,5,5’-Tetramethyl benzidine (TMB), which was colorless but would become blue oxTMB (character peak at 375 and 653 nm) after oxidation (Fig. 4a). Moreover, laser induced hyperthermia effect is expected to enhance the redox reaction. As shown in Fig. 4b, the characteristic peaks of oxTMB appeared in AgPd PB-containing solution (PBS) groups upon adding of H_2_O_2_ (pH 6.5), while being absent in the groups without AgPd PB nanozymes, indicative of the occurrence of Fenton-like reaction by AgPd PB nanozymes. Importantly, the intensity of absorbance peak from the AgPd + H_2_O_2_ + PBS group increased apparently after exposure to 1064 nm NIR laser, demonstrating the promoting effect of laser hyperthermia and LSPR effect. Michaelis-Menten steady-state kinetics was also utilized to investigate the enzyme activities of AgPd PB, where H_2_O_2_ (120, 100, 80, 60, 40 and 20 mM) was substrate. The absorbance of oxTMB solution at 653 nm was used to quantify the product amount through Beer-Lambert law (Eq. 2) (*ε* of 39 000 M^− 1^·cm^− 1^ for oxTMB) at each time point (Additional file 1: Fig. S6a and S6b), with derivative of product amount with respect to time yielding velocity. A Lineweaver–Burk plot was also utilized here to calculate *V*_max_ and *K*_m_; *V*_max_ and *K*_m_ of AgPd PB nanozyme were measured to be 2.49 × 10^− 8^ M·s^− 1^ and 37.86 mM without laser irradiation, and to be 5.42 × 10^− 8^ M·s^− 1^ and 33.15 mM under laser irradiation, exhibited a superior POD catalytic activity than some reported nanozyme as judged from the value of *K*_m_ and *V*_max_ (refer to Additional file 1: Table S3). The enlarged *V*_max_ and decreased *K*_m_ values clearly demonstrates the photoinduced apparent enhancement of catalytic capability of AgPd PB nanozyme (Fig. 4c and d). In addition, electron spin resonance (ESR) was utilized to further verify the •OH generation, in which 5,5-dimethyl-1pyrroline N-oxide (DMPO) was used to capture •OH, yielding an adduct of (DMPO-OH) that presented a characteristic ESR signal pattern. As shown in Fig. 4e, the apparent quadruple peaks with a specific intensity ratio of 1:2:2:1 demonstrates the existence of •OH. Notably, after 1064 nm laser irradiation, the peak shapes remained unchanged but the intensities showed a distinct enhancement, verifying that laser-induced heat and LSPR effect indeed can promote the production of •OH.

### In vitro nano-catalytic and cytotoxicity evaluation

**Fig. 5:**
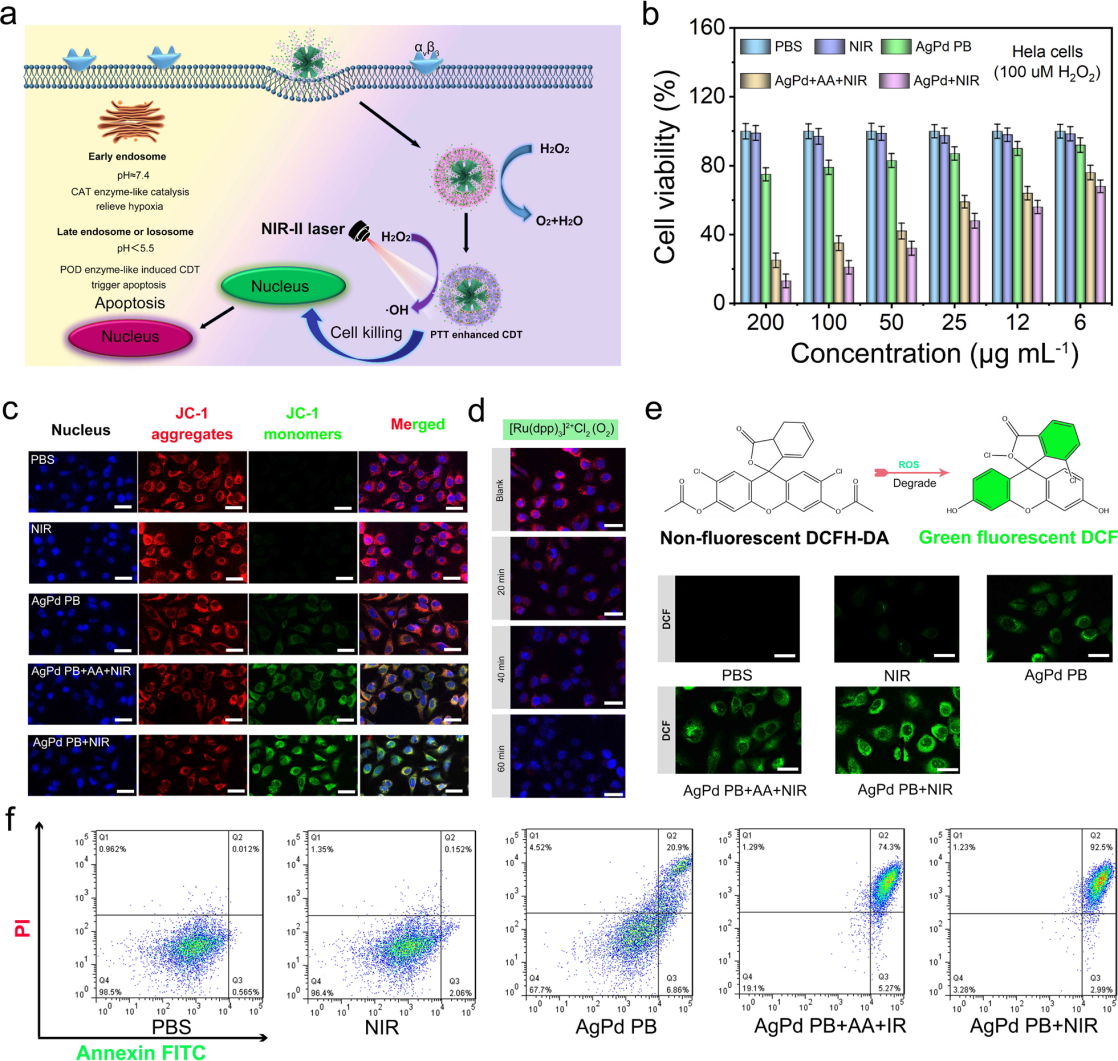
**a** Schematic illustration of subcellular pathways of AgPd PB nanozyme. **b** Cell viability of HeLa cells treated with different conditions (Control (1), NIR irradiation (2), AgPd PB (3), AgPd PB + AA + NIR (4) and AgPd PB + NIR (5)) *versus* the incubated particle concentration (25, 50, 75, 100, 150 and 200 µg·mL^-1^), mean ± s. d, n = 6. **c** The mitochondrial potential changes imaged by JC-1 staining under various conditions; Red JC-1 aggregate indicates mitochondria with a normal membrane potential, while the green JC-1 monomer represents the mitochondria with a depolarized membrane. **d** O_2_ generation test through catalysis of H_2_O_2_ (100 µM in aqueous solution) by AgPd PB nanozyme. **e** Detection of intracellular ROS after HeLa cells treated with different samples and conditions. **f** The flow cytometry of Hela cells treated with diverse conditions as that in (**b**)

The internalization process of AgPd PB nanozyme into HeLa cells was investigated using 4’,6-diamidino-2-phenylindole (DAPI) to label cell nucleus (blue emission), and fluorescein isothiocyanate (FITC, green emission) to covalently attach to AgPd PB nanozyme (Additional file 1: Fig. S7a). An elevated fluorescence signal of both blue and green were observed in whole-mount imaging of HeLa cells as well as in corresponding line-scan profiles at elapsed time, showing an effective accumulation of AgPd PB nanozyme in cell *via* endocytosis. The internalization process was schematized in Fig. 5a, in which AgPd PB nanozyme was first captured by an early endosome (pH ≈ 7.4) that presents CAT activity to relive tumor hypoxia. After translocating to the late endosome or lysosome (pH < 5.5), the acidic environment would promote AgPd PB nanozyme to exhibit POD activity, producing toxic •OH. Note that MTT assay results in Additional file 1: Fig. S7b after incubating AgPd PB nanozyme with L929 fibroblast cells for 24 and 48 h indicated a low toxicity of AgPd PB nanozyme to normal cells (above 87% viability with 48 h of culture, even at high concentration of 500 µg·mL^− 1^). Hemolysis experiment results also supported this, where hemolysis rate was measured to be lower than 5% for all investigated AgPd PB nanozyme concentrations (0-800 µg·mL^− 1^) (Additional file 1: Fig. S8). In contrast, AgPd PB nanozyme exhibit toxicity to HeLa cells, in particular under NIR light irradiation. The cytotoxicity to HeLa cells was investigated, by comparing cell viability (MTT method) in five groups (Control (1), NIR (2), AgPd PB nanozyme (3), AgPd PB nanozyme + NIR + AA (4), AgPd PB nanozyme + NIR (5)) at varying AgPd PB nanozyme concentrations (6-200 µg·mL^− 1^) (Fig. 5b). Vitamin c (AA) is an effective ROS scavenger, which was utilized here to eliminate •OH produced in POD enzymatic activities. As expected, 1064 nm laser produced negligible effects on group (1) and (2), while group (3) exhibited about 17% cell lethality at AgPd PB nanozyme maximum concentration (200 µg·mL^− 1^), being attributed to the POD activities of AgPd PB nanozyme to catalyze enriched H_2_O_2_ to •OH in tumor cells. For group (4) where •OH effect was removed, single PTT resulted in approximately 78% death of HeLa cells. Whereas for group (5) about 90% of HeLa cells were killed owing to the synergistic therapy of PTT and CDT. This conclusion was supported by direct observation of intracellular •OH (due to POD-activity of AgPd PB nanozyme), which can activate 7-hydroxycoumarin to produce blue emission (445 nm, excitation at 332 nm) (Additional file 1: Fig. S7c). Observation of increased blue fluorescence indicated accumulative production of •OH by AgPd PB nanozymes in tumor cells with prolonged incubation time.

Membrane-permeant JC-1 dye, widely used in cell apoptosis studies, was utilized here to detect the change of mitochondrial membrane potential (MMP). JC-1 dye exhibited potential-dependent accumulation in mitochondria, indicated by concentration-dependent formation J-aggregates with red fluorescence (~ 590 nm) (intact membrane) or by monomeric form with green fluorescence emission (~ 529 nm) (disruption of mitochondria membrane). As illustrated in Fig. 5c, strong red fluorescence was observed in group (1) and group (2) manifested negligible changes in mitochondrial membrane (negligible apoptosis). While for groups (3)–(5), intense green fluorescence was observed due to significant mitochondrial membrane potential transformation, indicating cell apoptosis. In particular, group (5) with AgPd PB nanozyme + laser have the highest green fluorescence, implying the most effective cell apoptosis. Moreover, intracellular O_2_ generation was also successfully demonstrated in AgPd PB nanozyme-containing HeLa cells, as O_2_-induced reduction of red fluorescence was observed in [Ru(dpp)_3_]Cl_2_ (RDPP) (Fig. 5d), thus giving direct evidence to the occurrence of AgPd PB nanozyme CAT activities in tumor cells. The amount of intracellular ROS was probed by non-fluorescent 2’-7’-dichorofuran-diacetate (DCFH-DA), which could be oxidized by ROS to exhibit green emission of 2’-7’-dichorofuorescin (DCF). As shown in Fig. 5e, the fluorescence was consistent with the observed intracellular ROS amount in HeLa cells, which was highest in group (5). Furthermore, flow cytometry with annexin V-FITC/PI double staining was performed investigate the apoptosis of HeLa cells (Fig. 5f). The apoptosis rate (containing both early and late apoptosis) for the five groups were measured to be 0.577%, 2.18%, 27.4%, 79.6% and 95.5%, respectively, consistent with the MTT results in Fig. 5b.

Taken together, we demonstrated that AgPd PB nanozymes were internalized to tumor cells through an endocytosis pathway and able to produce intracellular O_2_ through CAT activities and •OH through POD activities. And exposure to 1064 nm laser could bring a distinct acceleration to intracellular nanozyme activities, while the generated ROS (•OH) through CDT cooperated with PTT can induce efficient apoptosis of tumor cells.

### Imaging performance

With the advantages of high atomic number and strong attenuation to X rays (Ag with 47 and Pd with 46 of atomic number), AgPd PB nanozyme was investigated as computed tomography (CT) contrast agents for in vivo and in vitro bioimaging. The Hounsfield unit (HU) of CT signal are proportional to of AgPd PB nanozyme concentrations with a slope of 5.622, showing enhanced image contrast at higher concentrations (Additional file 1: Fig. S9a and b). Afterwards, the in vivo CT imaging was implemented on tumor-bearing mice after intratumorally injection of AgPd nanozyme. As presented in Additional file 1: Fig. S9c–f, there was a significant CT signal effect at the tumor site in comparison with the control group, demonstrating the potential use of AgPd nanozyme as CT contrast agents.

**Fig. 6:**
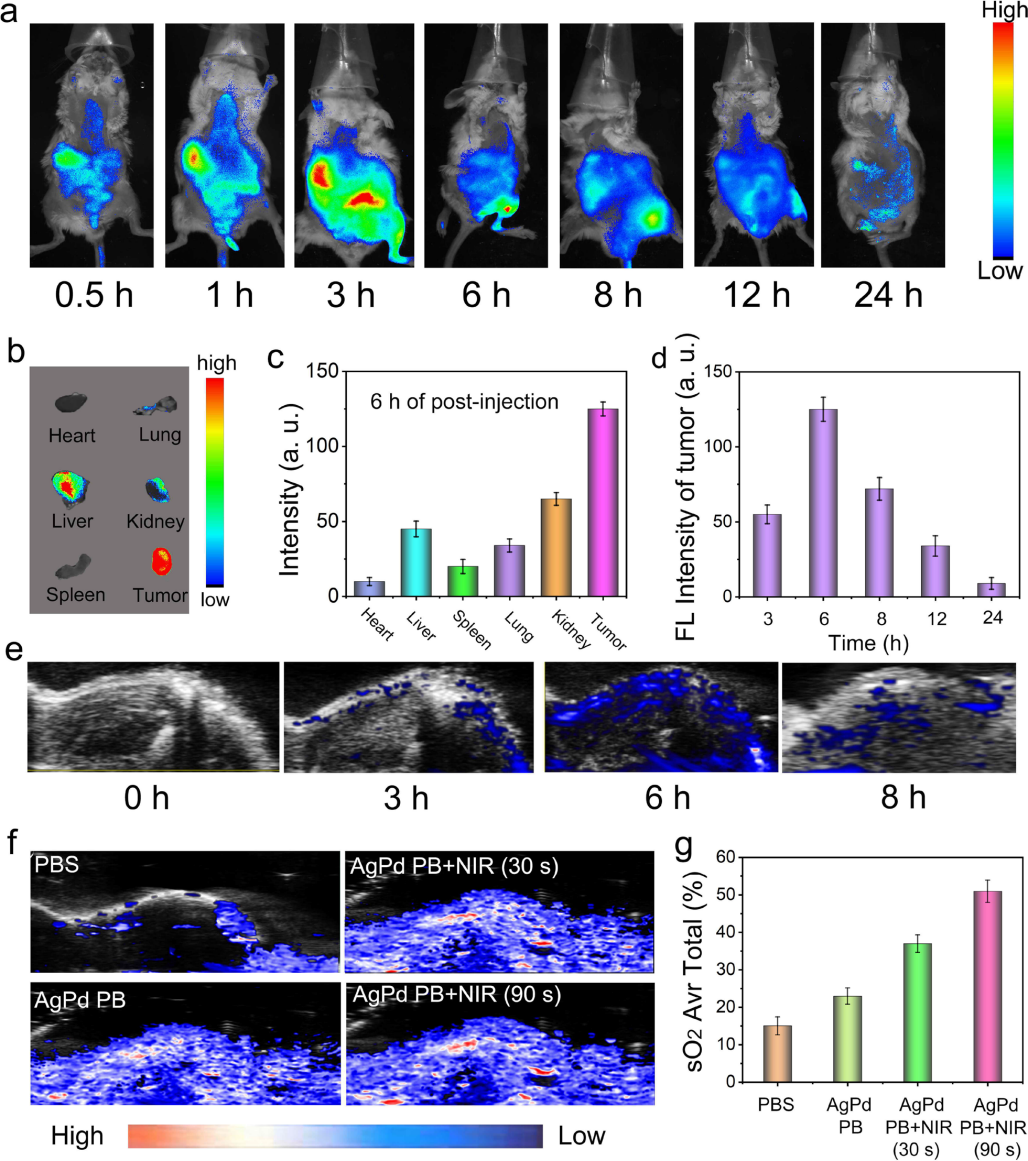
**a** In vivo fluorescence images of stained AgPd PB nanozyme at different time points post IV injection. **b** In vivo fluorescence images of harvested major organs and tumors of mice sacrificed at 6 h post IV injection, with relative fluorescence intensity shown in **c**. **d** The fluorescence intensity of tumor at diverse time points post IV injection. **e** Tumor photoacoustic imaging of tumor-bearing mice after injected with AgPd PB nanozyme at different time points. **f** Photoacoustic imaging of tumor blood O_2_ with the estimated O_2_ content in tumor area shown in **g**

Since AgPd PB nanozyme is unable to fluorescence, rhodamine B (RB, excitation at 540 nm, emission at 625 nm) was conjugated with AgPd PB nanozyme to allow fluorescence tracking. Fluorescence of RB-stained AgPd PB nanozyme has a regular digital image change in intensity (Additional file 1: Fig. S10a), exhibited a linear correlation with the nanoparticle concentrations (Additional file 1: Fig. S10b). The mouse cervical cancer cell (U14) tumor-bearing Kunming mice were intravenously injected with RB-stained AgPd PB nanozyme, and time-dependent biodistribution of AgPd PB nanozyme was investigated. As shown in Fig. 6a, after 1 h of injection, fluorescence appeared in liver and kidney with weak fluorescence observed in the tumor site. The fluorescence signal at tumor site reached a maximum at 6 h post-injection, indicating the highest tumor accumulation of AgPd PB nanozyme, possibly due to the enhanced permeability and retention (EPR) effect. After this, decaying of fluorescence in both tumor and liver areas was observed at 8 and 12 h of injection. After 24 h of post-injection, almost all the fluorescence disappeared, implying the clearance of nanozyme from body. At 6 h of post injection, tumors and main major internal organs from euthanized mice were harvested presenting fluorescence in liver, tumor, kidney, and lung (Fig. 6b), with the highest one observed in tumor (Fig. 6c). Plotting fluorescence from tumor against time demonstrated a maximum accumulation of AgPd nanozyme in the tumor, signifying the optimal timing for the treatment (Fig. 6d). To quantitatively evaluate the distribution and to probe metabolic pathways of AgPd PB nanozyme, an inductively coupled plasma emission spectrometer (ICP-OES) was utilized to measure Pd element content in major organs (from left to right as the heart, liver, spleen, lung, kidney), urine and feces after dissecting mice at 1, 3 and 5 days. As displayed in Additional file 1: Fig. S11b and c, after 5 days of intravenous injection, only a hint amount of AgPd PB nanozyme (less than 2.8 ID %/g) remained in liver and spleen, while no obvious existence was identified in other organs. Moreover, over 90% AgPd PB nanozyme were observed to be cleared from the body through urine (10%) and feces (80%), meaning an efficient excretion through both kidney and liver pathways in a short period of time. Hematoxylin and eosin (H&E) staining was performed to investigate anatomic structures of major internal organs (heart, kidneys, liver, lung and spleen) (Additional file 1: Fig. S13), in which no obvious pathological changes were observed, indicating the low toxicity of AgPd PB nanozyme to normal tissues.

Photoacoustic (PA) imaging is an emerging imaging method to monitor the retention effect of the material in tumor. As presented in Fig. 6e, PA imaging of tumor were visualized after 0/3/6/8 h intravenous injection of AgPd PB nanozyme. It can be observed that the material began to enrich at the tumor site at 3 h, reach a maximum enrichment at 6 h and then gradually cleared after 8 h of injection, consistent with the results of fluorescence in vivo.

The distribution of O_2_ in living systems can be achieved by comparing PA signals utilizing specific wavelengths of light corresponding to oxygenated hemoglobin (HBO) and deoxygenated hemoglobin (Hb). Comparing with PBS control group, tumor that contains AgPd PB nanozyme clearly shows the existence of O_2_ (Fig. 6e). Moreover, exposure to 1064 nm laser resulted in an instantaneous increase of O_2_ content, and prolonging laser irradiation induced higher O_2_ content production (Fig. 6f). The O_2_ content in the tumor region was increased from 15 to 52%, demonstrating the ability of AgPd PB nanozyme for alleviating tumor hypoxia in vivo.

### Antitumor efficacy in vivo of AgPd PB nanozyme

Encouraged by in vitro therapeutic effects, we then evaluated AgPd PB nanozyme anti-tumor performance in vivo. After 7 days of subcutaneous inoculation of mouse cervical cancer cells (U14) to the right hind leg, tumor-bearing Kunming mice model were accomplished for in vivo experiments. All tumor-bearing mice were randomly divided into five groups (n = 5) to perform five distinct treatments (PBS as control, 1064 nm laser irradiation (NIR), AgPd PB nanozyme (AgPd PB), AgPd PB nanozyme with AA plus 1064 nm laser irradiation (AgPd PB + AA + NIR) and AgPd PB nanozyme plus 1064 nm laser irradiation (AgPd PB + NIR)).

**Fig. 7:**
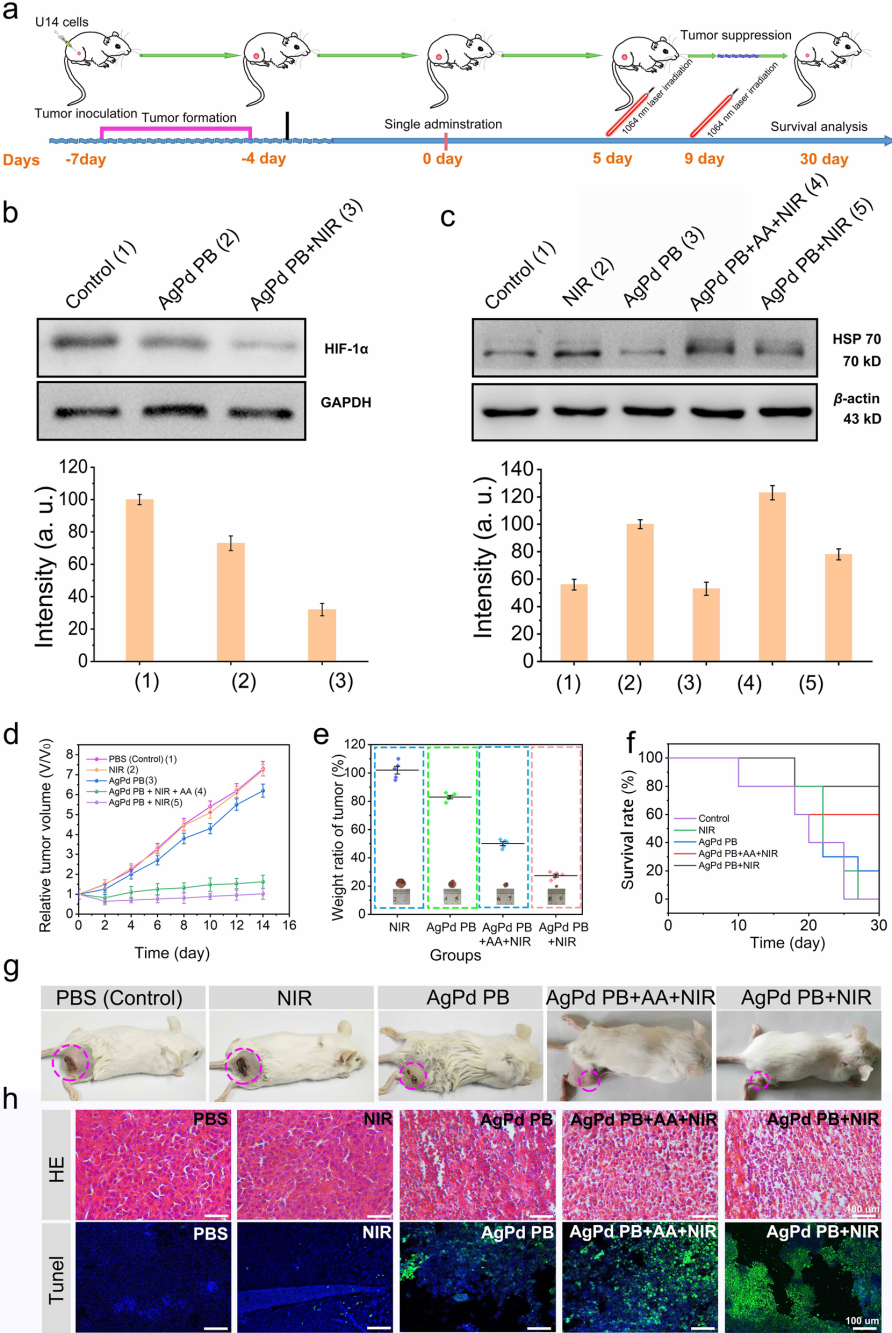
**a** Chronicle schematic depict of the establishment of U14 tumor xenograft model, administration of AgPd PB nanozyme, laser treatment, and analysis of therapeutic outcome. Western blots of **b** HIF-α and **c** HSP 70. **d** The tumor growth curves with diverse treatments. **e** Proportion of tumor weight in various groups relative to that in untreated mice obtained after 21 days of treatment. **f** The survival curve of mice after different treatments. **g** Representative digital images of the mice under different treatments after 21 days of treatments. **h** H&E-staining and TUNEL photographs of tumor slices obtained from tumor-bearing mice treated with various treatments. The error bars are based on the standard errors of the mean (n = 5). Scale bars: 100 μm

Western blotting assays was used to evaluated the levels of hypoxia-induced factor (HIF-1a) protein and HSP 70 in tumor cells. Compared with the control group, HIF-1a protein level was lower in AgPd PB group, which was further decreased in the AgPd PB + NIR group. This observation indicated that the AgPd PB nanozyme can alleviate hypoxia in tumor cells in vivo, in particular, under laser irradiation (Fig. 7b top of WB images and bottom of intensity). Besides, compared with the control group, the HSP 70 level in NIR irradiation group was up-regulated, whereas a down-regulation was observed in the AgPd PB group (Fig. 7c top of WB images and bottom of intensity), indicating the inhibitory effect of ROS on HSP 70 protein expression. This was also verified by the observation of higher HSP 70 content in AgPd PB + AA + laser group than in AgPd PB + laser group, in which ROS was cleared by AA in the former group. This result indicated that AgPd PB produced ROS is potent to suppress HSP 70 protein in vivo, thus favoring PTT effect.

After intravenous injection of AgPd PB nanozyme (5 mg·mL^− 1^, 100 µL) for 6 h, we then implemented 1064 nm near-infrared laser irradiation treatment with a power density of 1.2 W·cm^− 2^ for 5 min at day 5 and day 9 (Fig. 7a). Note that under such laser exposure the temperature of tumor could ascend from room temperature to about 47 ℃, indicating the preeminent PTT features of AgPd PB nanozyme (Additional file 1: Fig. S12). Tumor analysis and recording were then carried out for next consecutive 14 days. Digital graphs of tumor-bearing mice were taken for different groups after treatment, with the smallest tumor was observed for mice group treated with AgPd PB + NIR (Fig. 7 g). Indeed, the 5 mice treated with AgPd PB + NIR always exhibited the smallest tumor volume and the lowest tumor weight ratio over the 14 day observation period (Fig. 7d and e), proving that the AgPd PB + NIR combination was able to produce a strong synergistic CDT and PDT antitumor therapeutic effect.

At 14 days post-treatment, the group of AgPd + NIR showed the highest survival rate (80%) (Fig. 7f). Biochemical analysis and blood analysis (Additional file 1: Fig. S13 a-h) of the control group and the material treatment group at different time points (1, 7, 14 days), together with histology staining images of major organs of mice (Additional file 1: Fig. S13i) indicated that AgPd PB nanozyme presents no apparent toxicity. Furthermore, H&E stained tumor tissues indicated the mice treated with AgPd PB + NIR shown the most severe cell apoptosis, demonstrating the effective therapeutics of combined PTT and CDT (Fig. 7 h top). TUNEL (terminal deoxynucleotidyl transferase nick end labeling) assays were also performed to investigate apoptosis in tumor tissue harvested from mice under diverse group treatment (Fig. 7 h, bottom). DAPI and TUNEL were utilized to stain nucleus (DAPI, blue) and cytoplasm (TUNEL, green). TUNEL detects the DNA breaks formed when DNA fragmentation occurs in apoptosis, respectively. It can be clearly seen that the green fluorescence (representing the apoptotic issue) gradually increases from the AgPd PB group to the AgPd PB + NIR group, again confirming that the synergistic CDT and PTT (from the AgPd PB + NIR group) results in the most effective tumor cell apoptosis.

## Conclusion

In summary, we have successfully developed a type of biomimetic plasmonic AgPd PB nanozyme with a compact size of 30 nm, which can produce synergistic CDT and PTT for effective tumor treatment under tissue-penetrating light irradiation. An intense and broadband LSPR absorbance across 400–1300 nm empowered efficient photothermal conversion with an efficiency of 45.1% under NIR-II irradiation at 1064 nm. Moreover, plasmonic AgPd PB nanozyme exhibited both CAT (through Eley–Rideal mechanism) and POD-mimic activities (through Fenton-like mechanism), which were demonstrated to catalyze TME-enriched H_2_O_2_ to into O_2_ to relieve tumor hypoxia or into toxic •OH to induce apoptosis in vitro and in vivo. Moreover, we demonstrated that laser irradiation at 1064 nm elevated maximum catalytic velocity (*V*_max_) and reduced Michaelis–Menten constant (*K*_m_) of AgPd PB nanozyme, showing boosted nanozyme efficacy to induce more efficient HeLa cell apoptosis. Moreover, POD-mimic activities produced ROS (toxic •OH) can down-regulate HSPs (which inhibit hyperthermia), favoring PTT treatment. In faith, laser-activated AgPd PB nanozyme were able to deliver efficient synergistic CDT and PTT treatment, which minimizes tumor size and weight and presents the highest survival rate. Along the shown CT, fluorescence, and PA imaging abilities as well as an efficient clearance from body, the plasmonic AgPd PB nanozymes described here hold great promises for precise tumor theranostics.

## Supplementary Information

**Additional file 1: Fig. S1**. Zeta potentials and dynamic light scattering (DLS) of AgPd PB.** Fig. S2**. XRD patterns, EDS spectrum and XPS spectrum of AgPd PB nanozyme.** Fig. S3**. Mass extinction coefficient and photothermal imaging of AgPd PB nanozyme.** Fig. S4**. The comparison of the effect of LSPR and PPT to CAT and POD enzyme activities.** Fig. S5**. The O_2_ generation of H_2_O_2_ incubated with different concentration of AgPd PB nanozyme without laser irradiation and the relative catalytic activity of AgPd PB nanozyme.** Fig. S6**. Time-course absorbance of AgPd PB nanozyme upon the addition of varied concentrations of H_2_O_2_ (120, 60, 30 and 15 mM) without and with laser irradiation. **Fig. S7**. phagocytosis, biological activity and ROS assay of AgPd PB nanozyme.** Fig. S8**. Hemolysis assay for AgPd PB nanozyme.** Fig. S9**. In vitro and in vivo CT images of AgPd PB nanozyme.** Fig. S10**. Fluorescent values of AgPd nanozyme in accordance with concentrations and the correlated linear fitting.** Fig. S11**. Schematic illustration of the body’s rapid clearance of AgPd PB nanozyme.** Fig. S12**. IR thermal images at the tumor sites of U14-tumor-bearing mice under 1064 nm laser (1.25 W·cm^− 2^) irradiation with saline and AgPd PB nanozyme at different time intervals.** Fig. S13**. H&E stained tissue images of heart, liver, spleen, lung, and kidney excised from mice after 14 days of treatment of AgPd PB nanozyme.** Table S1**. Comparison of properties between some other photothermal materials with AgPd PB nanozyme.** Table S2**. The photothermal conversion efficiency (PTCE) of molar extinction coefficient (MEC) of several PTT agents.** Table S3**. The Michaelis-Menton constant (*K*_m_) and maximum reaction rate (*V*_max_) of previously reported nanozyme with H_2_O_2_ as the substrate for POD-mimic catalysis.

## Data Availability

The datasets and materials used in the study are available from the corresponding author.

## Declarations

(Beijing, China) (1100111084356). All mouse experiments conform to the standards of The National Regulation of China for Care and Use of Laboratory Animals, and have been approved by the Ethics Committee of the Second Affiliated Hospital of Harbin Medical University (SYDW 2019–82).
