# Supplementary material for: A bimodal type of AgPd Plasmonic Blackbody Nanozyme with boosted catalytic efficacy and synergized photothermal therapy for efficacious tumor treatment in the second biological window
Source: J Nanobiotechnology. 2022 Sep 24;20:424. doi: 10.1186/s12951-022-01627-y (PMC9509650; doi:10.1186/s12951-022-01627-y)
Supplement: Supplementary file 1 — Additional file 1: Fig. S1. Zeta potentials and dynamic light scattering (DLS) of AgPd PB. Fig. S2. XRD patterns, EDS spectrum and XPS spectrum of AgPd PB nanozyme. Fig. S3. Mass extinction coefficient and photothermal imaging of AgPd PB nanozyme. Fig. S4. The comparison of the effect of LSPR and PPT to CAT and POD enzyme activities. Fig. S5. The O2 generation of H2O2 incubated with different concentration of AgPd PB nanozyme without laser irradiation and the relative catalytic activity of AgPd PB nanozyme. Fig. S6. Time-course absorbance of AgPd PB nanozyme upon the addition of varied concentrations of H2O2 (120, 60, 30 and 15 mM) without and with laser irradiation. Fig. S7. phagocytosis, biological activity and ROS assay of AgPd PB nanozyme. Fig. S8. Hemolysis assay for AgPd PB nanozyme. Fig. S9. In vitro and in vivo CT images of AgPd PB nanozyme. Fig. S10. Fluorescent values of AgPd nanozyme in accordance with concentrations and the correlated linear fitting. Fig. S11. Schematic illustration of the body’s rapid clearance of AgPd PB nanozyme. Fig. S12. IR thermal images at the tumor sites of U14-tumor-bearing mice under 1064 nm laser (1.25 W·cm− 2) irradiation with saline and AgPd PB nanozyme at different time intervals. Fig. S13. H&E stained tissue images of heart, liver, spleen, lung, and kidney excised from mice after 14 days of treatment of AgPd PB nanozyme. Table S1. Comparison of properties between some other photothermal materials with AgPd PB nanozyme. Table S2. The photothermal conversion efficiency (PTCE) of molar extinction coefficient (MEC) of several PTT agents. Table S3. The Michaelis-Menton constant (Km) and maximum reaction rate (Vmax) of previously reported nanozyme with H2O2 as the substrate for POD-mimic catalysis. [file 12951_2022_1627_MOESM1_ESM.docx]

**A Bimodal Type of AgPd Plasmonic Blackbody Nanozyme with Boosted Catalytic Efficacy and Synergized Photothermal Therapy for Efficacious Tumor Treatment in the Second Biological Window**

Tao Jia,^1^ Dan Li,^1^ Jiarui Du,^1^ Xikui Fang,^1^ Valeriy Gerasimov,^1^ Hans Ågren,^1^ and Guanying Chen^1,^*

^1^MIIT Key Laboratory of Critical Materials Technology for New Energy Conversion and Storage, School of Chemistry and Chemical Engineering & State Key Laboratory of Urban Water Resource and Environment & Key Laboratory of Micro-systems and Micro-structures Ministry of Education, Harbin Institute of Technology, Harbin, 150001, P. R. China

*Correspondence: [chenguanying@hit.edu.cn](mailto:chenguanying@hit.edu.cn)


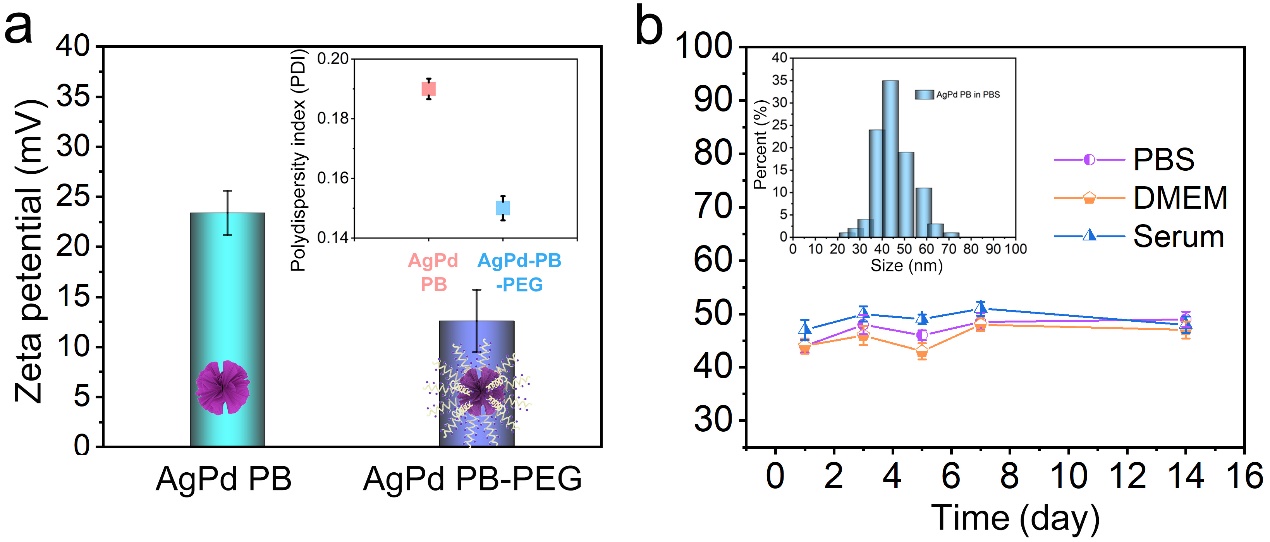


**Fig. S1** (**a)** Zeta potentials of AgPd PB and AgPd PB-PEG. The particle size distributions of AgPd PB-PEG in water measured by dynamic light scattering (DLS) (inset) and (**b)** the particle size distributions of the supernatant obtained from the AgPd PB-PEG solutions in culture medium, serum and PBS (pH 7.4) and after two weeks standing.


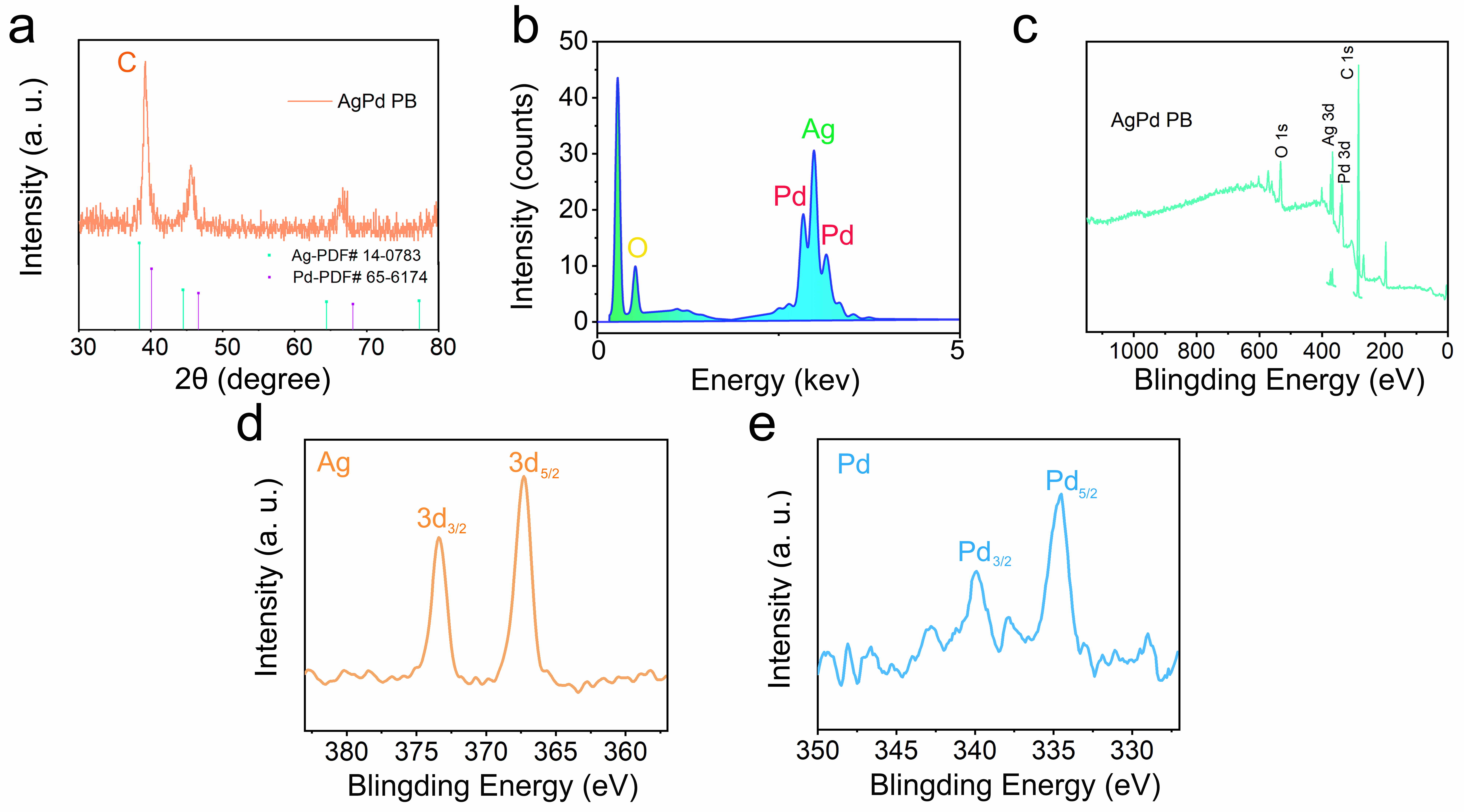


**Fig. S2 (a)** XRD patterns, (**b)** EDS spectrum and (**c)** XPS spectrum and corresponding **(d)** Ag 3d and **(e)** Pd 3d region of as-synthesized AgPd PB nanozyme.


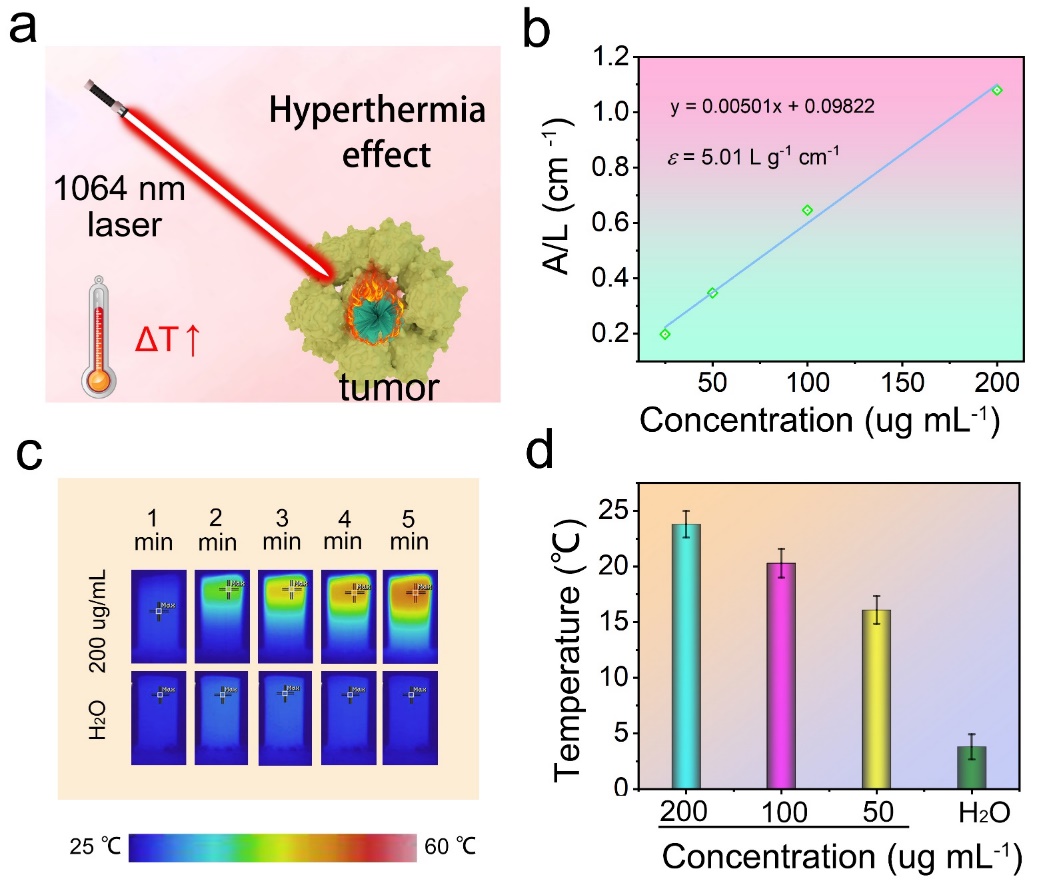


**Fig. S3 (a)** The illustration of AgPd PB nanozyme induced photothermal therapy at excitation of 1604 nm laser. (**b)** Mass extinction coefficient of AgPd PB nanozyme at 1064 nm. Normalized absorbance intensity at *λ*=1064 nm measured by the characteristic length of the cell (A/L) at varied concentrations (25, 50, 100, and 200 mg mL^-1^). (**c)** Photothermal imaging of AgPd PB nanozyme within min and (**d)** temperature change of AgPd PB nanozyme of various concentrations over a period of 300 s.


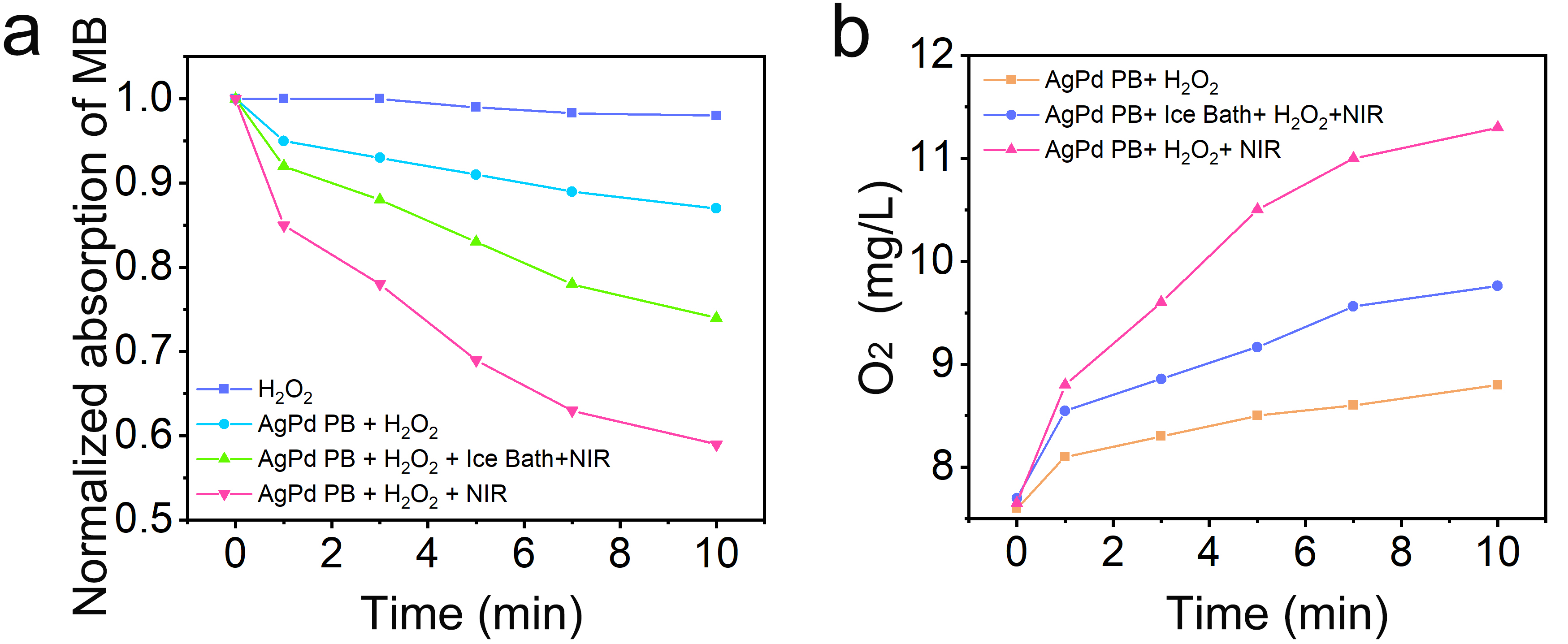


**Fig. S4** **(a)** The normalized UV-Vis absorbance of MB treated with NIR+ H_2_O_2_, AgPd PB + H_2_O_2_, AgPd PB + H_2_O_2_+ Ice Bath + NIR and AgPd PB + H_2_O_2_ + NIR. **(b)** O_2_ generation test through catalysis of H_2_O_2_ (100 µM in aqueous solution with pH 6.5) treated with treated with AgPd PB + H_2_O_2_, AgPd PB + H_2_O_2_+ Ice Bath + NIR and AgPd PB + H_2_O_2_ + NIR.


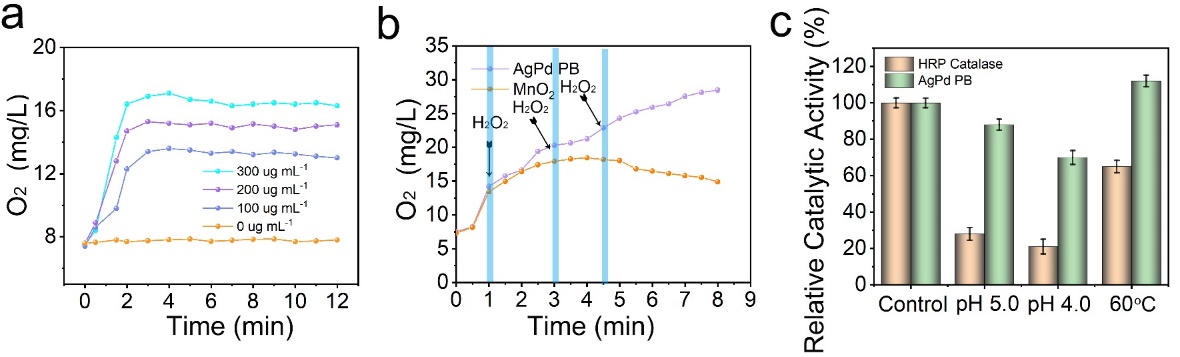


**Fig. S5** **(a)** The O_2_ concentration of H_2_O_2_ incubated with different concentration of AgPd PB nanozyme without laser irradiation. (**b)** The O_2_ concentration of H_2_O_2_ incubated with different concentration of AgPd PB nanozyme and under irradiation by 1064 nm laser from 5th min to 10th min. (**c)** The relative catalytic activity of AgPd PB nanozyme and HRP catalase under diverse conditions.


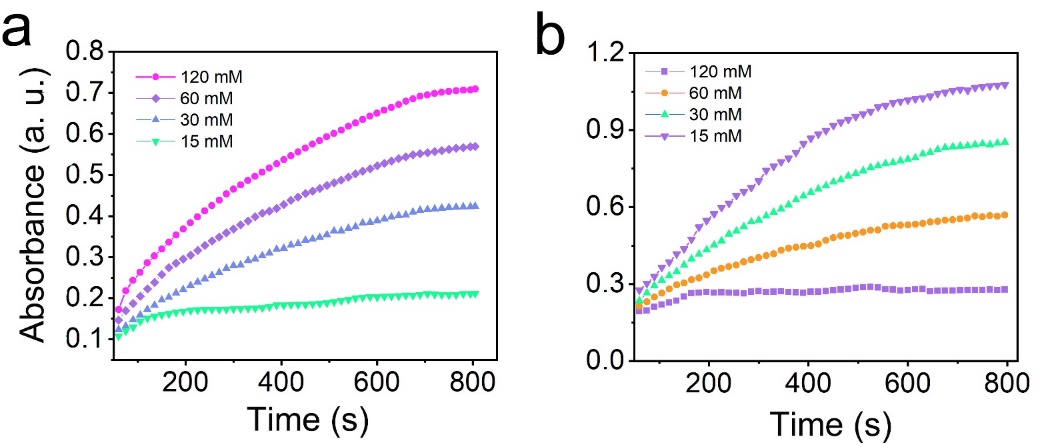


**Fig. S6** Time-course absorbance of AgPd PB nanozyme upon the addition of varied concentrations of H_2_O_2_ (120, 60, 30 and 15 mM) (**a)** without and (**b)** with laser irradiation (d).


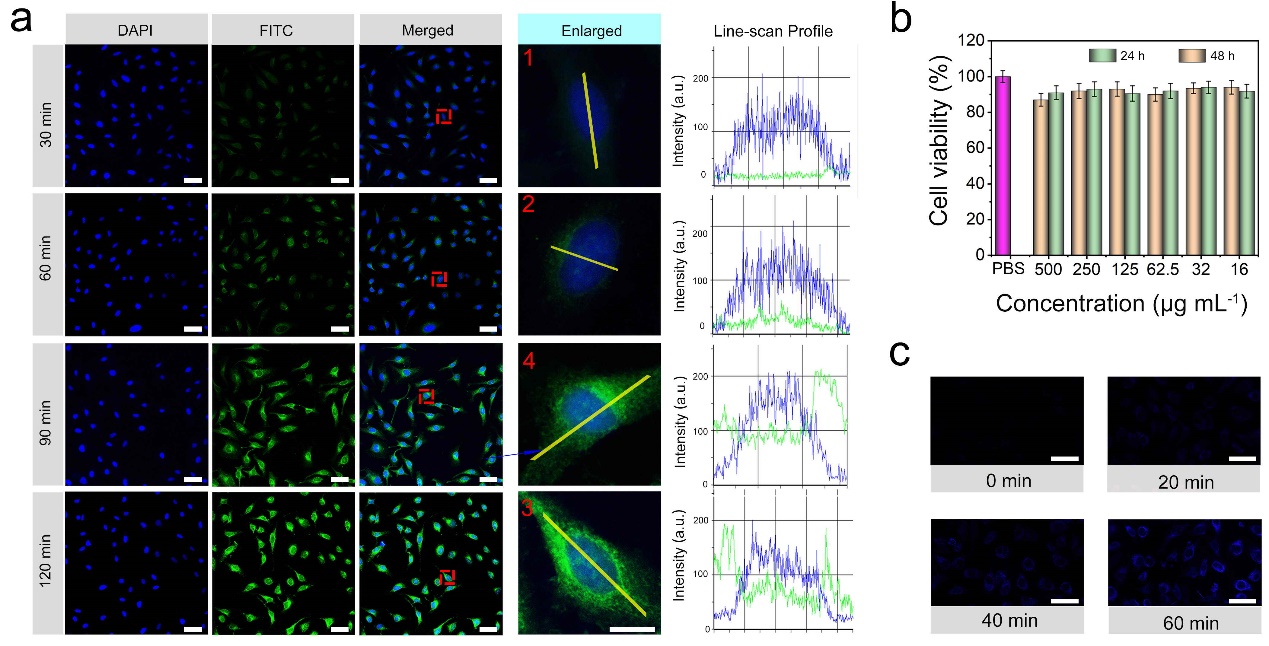


**Fig. S7** **(a)** CLSM images of HeLa cells dyed with DAPI and incubated with FITC-conjugated AgPd PB nanozyme and the corresponding line-scan profiles at different time intervals (30, 60, 90 min). (**b)** Cell viabilities of L929 fibroblast cells of 24 h and 48 h incubation with different concentration of AgPd PB nanozyme. (**c)** Intracellular •OH detection by coumarin. Scale bar: 50 µm.


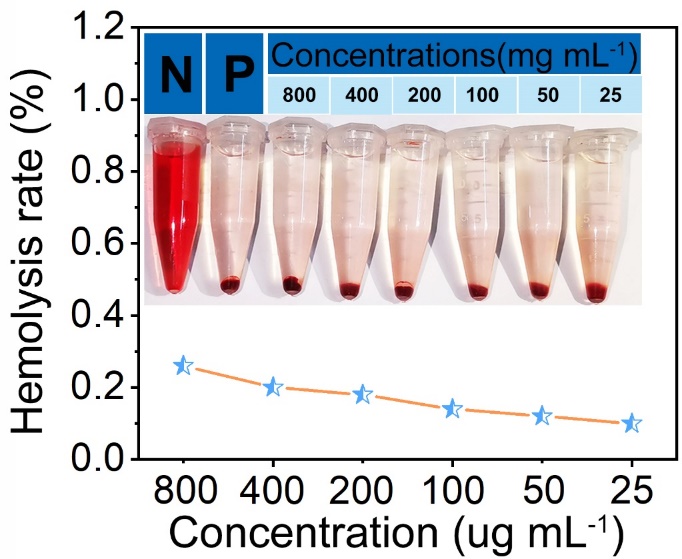


**Fig. S8** Hemolysis assay for AgPd PB nanozyme (inset: photographic images for direct observation of hemolysis by AgPd PB nanozyme using PBS as a negative control and water as a positive control (left two tubes), and AgPd PB nanozyme suspensions with different concentrations)


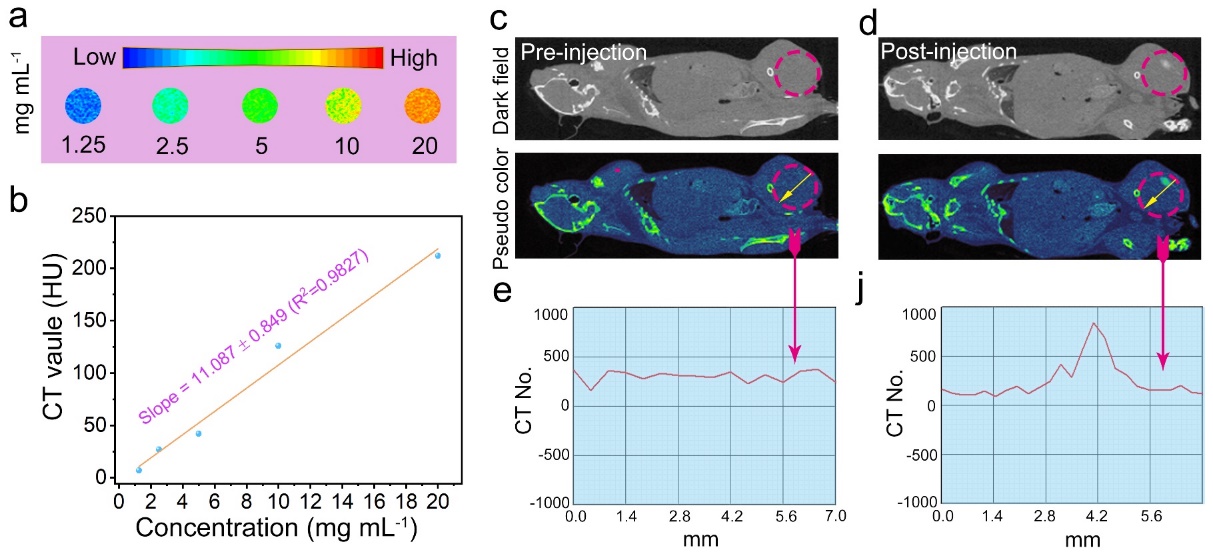


**Fig. S9 (a)** *In vitro* CT images of AgPd PB nanozyme with different concentrations. **(b)** The relationship between CT value of AgPd PB nanozyme aqueous solution. **(c-f)** CT images of tumor-bearing mice before and after AgPd PB nanozyme injection and the corresponding cross-sectional compositional line profiles of CT value.


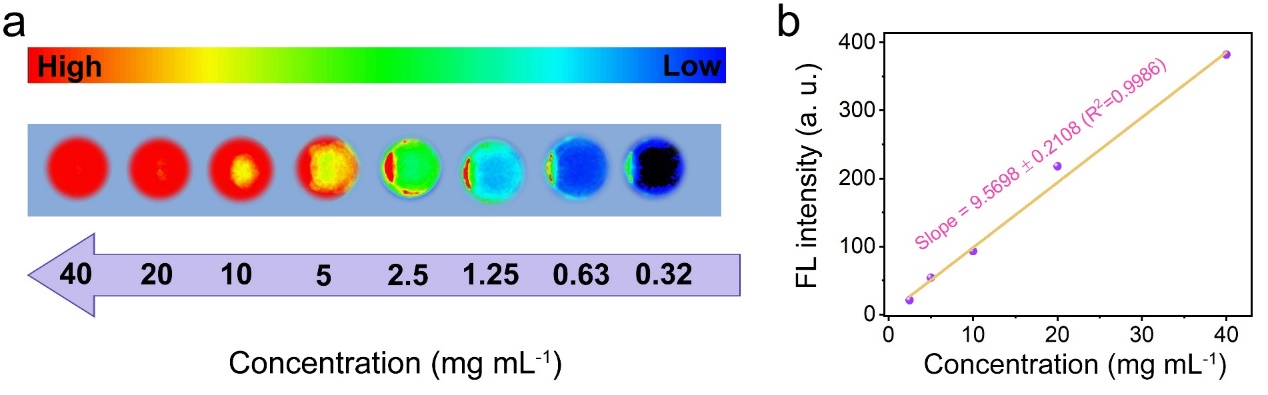


**Fig. S10** **(a)** Fluorescent values of AgPd nanozyme in accordance with concentrations and **(b)** the correlated linear fitting


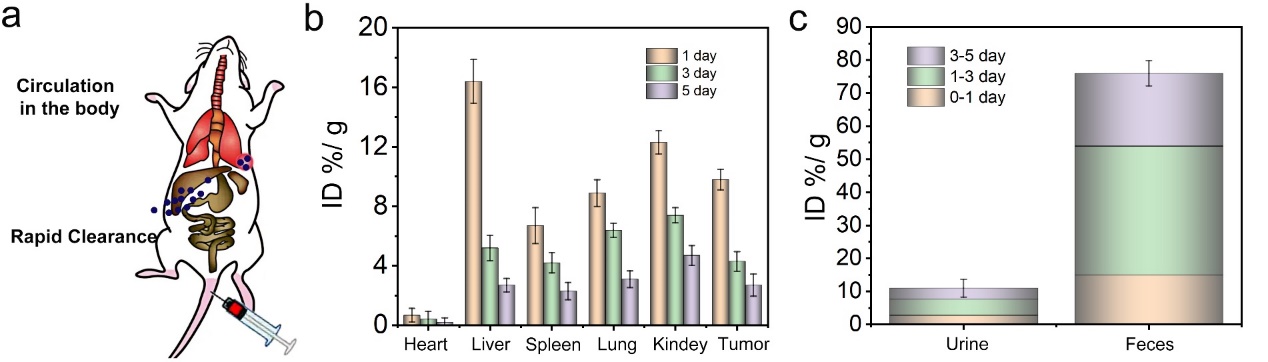


**Fig. S11** **(a)** Schematic illustration of the body’s rapid clearance of AgPd PB nanozyme. **(b)** Ag concentration in main organs and **(c)** in urine and feces of mice of mice at different time points.


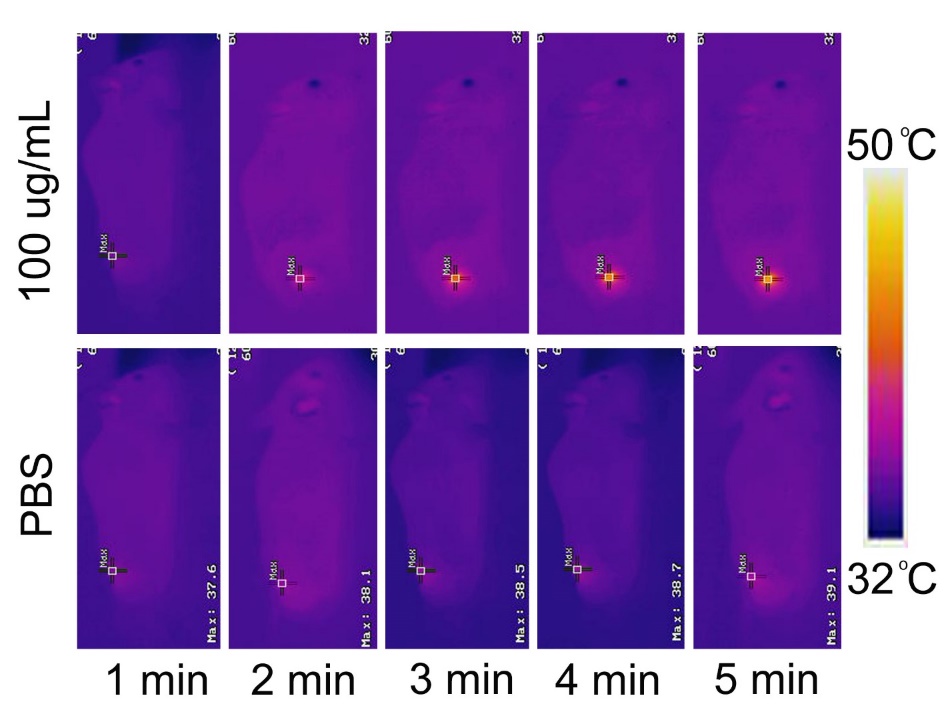


**Fig. S12** IR thermal images at the tumor sites of U14-tumor-bearing mice under 1064 nm laser (1.25 W·cm^−2^) irradiation with saline and AgPd PB nanozyme at different time intervals.


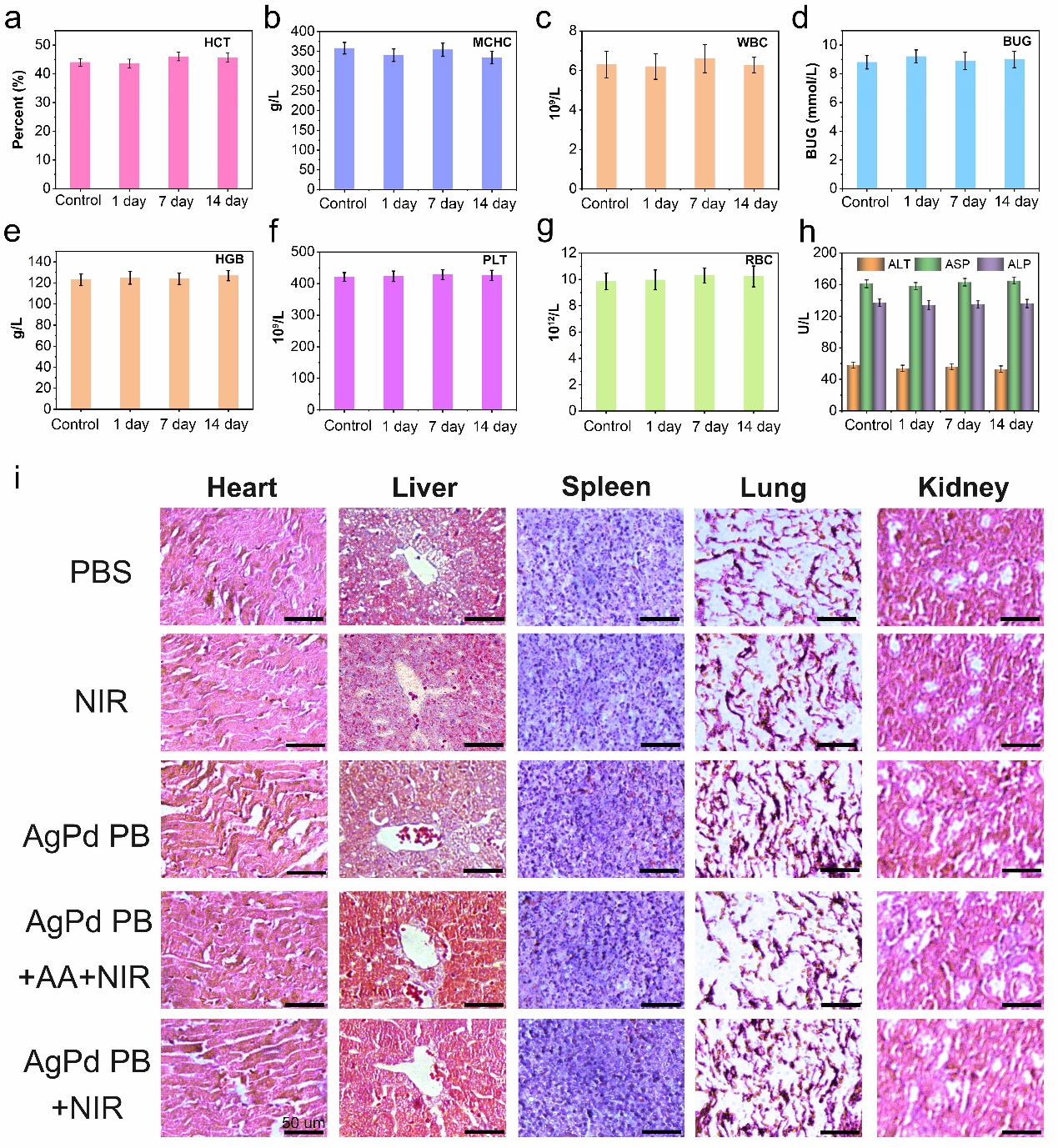


**Fig. S13.** The blood biochemistry and hematology data of female Kunming mice treated with UCSA-PEG in different times were as follow: **(a)** hematocrit (HCT), **(b)** mean corpuscular hemoglobin concentration (MCHC), **(c)** white blood cells (WBC), **(d)** blood urea nitrogen (BUN) levels, **(e)** hemoglobin (HGB), **(f)** platelets (PLT), **(g)** red blood cells (RBC) and **(h)** liver function indicators. **(i)** Histology staining images of major organs of mice with different treatments.

**Table S1.** Comparison of properties between some other photothermal materials with AgPd PB nanozyme.

| **Material （diameter）** | **PTCE (%)** | **Excitation Wavelength** | **Enzyme activity** | **Therapy** | **References** |
| --- | --- | --- | --- | --- | --- |
| Au nanostar  (126 ± 9 nm) | 36.4% | 1064 nm | N.A. | PTT/radiotherapy | ACS applied materials & interfaces, 12 (2020) 36928-36937 |
| Cu_9_S_8_ (100 nm)/ | N.A. | N.A. | POD | PTT/CDT  synergized therapy | 10.1016/j.nantod.2022.101397 |
| AgPd alloy (28 nm) | 45.1% | 1064 nm | CAT/POD | PTT/CDT synergized therapy | **This work** |
| CuFe nanospheres (70 nm)/ | 27.8% | 808 nm | CAT | PTT/PDT/CDT  synergized therapy | ACS nano, 12 (2018) 4886-4893 |
| FePd (50 nm)/ | 35.4% | 1064 nm | N.A. | PTT/radiotherapy | Biomaterials, 233 (2019) 119656 |

**Table S2.** The photothermal conversion efficiency (PTCE) of molar extinction coefficient (MEC) of several PTT agents.

| **Material（diameter）** | **PTCE (%)** | **Excitation Wavelength** | **MEC（**M^-1^ cm^-1^**）** | **References (DOI)** |
| --- | --- | --- | --- | --- |
| Au-Cu_9_S_5_ (100 nm) | 37% | 1064 nm | 10^8^ | [10.1021/ja508641z](https://doi.org/10.1021/ja508641z) |
| Fe_3_O_4_@CuS (100 nm) | 19.2% | 1064 nm | 10^10^ | [10.1002/adfm.201503015](https://doi.org/10.1002/adfm.201503015) |
| AgPd alloy (28 nm) | 45.1% | 1064 nm | 10^9^ | **This work** |
| CuS | 26.7% | 1064 nm | / | [10.1021/acsami.9b23413](https://doi.org/10.1021/acsami.9b23413) |
| Cu_3_BiS_3_ (50 nm) | 40.7% | 1064 nm | / | [10.1016/j.biomaterials.2016.10.024](https://www.x-mol.com/paperRedirect/1212911219176710149) |
| Pd@Au (30 nm) | 32.3% | 1064 nm | / | 10.1002/ange.201906758 |

**Table S3.** The Michaelis-Menton constant (*K*_m_) and maximum reaction rate (*V*_max_) of previously reported nanozyme with H_2_O_2_ as the substrate for POD-mimic catalysis.

| nanozyme | *V*_max_ (M s^–1^) | *K*_m_ (mM) | reference |
| --- | --- | --- | --- |
| HRP | 0.69 × 10^–9^ | 10.35 | [S1] |
| Naked-Fe_3_O_4_ | 3.06 × 10^–9^ | 458,9 | [S1] |
| Ala-Fe_3_O_4_ | 4.45 × 10^–9^ | 226.6 | [S1] |
| His-Fe_3_O_4_ | 5.28 × 10^–9^ | 37.99 | [S1] |
| PtFe | 8.182 × 10^–8^ | 217.6 | [S2] |
| Fe_2_O_3_ | 3.05 × 10^–8^ | 86.43 | [S3] |
| Carbon nanohorn | 2.07 × 10^–8^ | 49.8 | [S4] |
| Zn-CuO | 3.0 × 10^–9^ | 71 | [S5] |
| Pt | 2.2 × 10^–12^ | 84.07 | [S6] |
| CoAl-ELDH | 5.98 × 10^–9^ | 22.13 | [S7] |

[S1] K. Fan, H. Wang, J. Xi, Q. Liu, X. Meng, D. Duan, L. Gao, X. Yan, *Chem. Commun.* **2017**, 53: 424-27.

[S2] S. Li, L. Shang, B. Xu, S. Wang, K. Gu, Q. Wu, Y. Sun, Q. Zhang, H. Yang, F. Zhang, L. Gu, T. Zhang, H. Liu, *Angew. Chem., Int. Ed.* **2019**, 58: 12624-31.

[S3] S. Tanaka, Y. V. Kaneti, R. Bhattacharjee, M. N. Islam, R. Nakahata, N. Abdullah, S.-i. Yusa, N. Nam-Trung, M. J. A. Shiddiky, Y. Yamauchi, M. S. A. Hossain, *ACS Appl. Mater. Interfaces* **2018**, 10: 1039-49.

[S4] Z. Wang, X. Lv, J. Weng, *Carbon* **2013**, 62: 51-60.

[S5] A. P. Nagvenkar, A. Gedanken, *ACS Appl. Mater. Interfaces* **2016**, 8: 22301-08

[S6] J.-M. Park, H.-W. Jung, Y. W. Chang, H.-S. Kim, M.-J. Kang, J.-C. Pyun, *Anal. Chim. Acta* **2015**, 853:360-67.

[S7] X. Wei, J. Chen, M. C. Ali, J. C. Munyemana, H. Qiu, *Microchim. Acta* **2020**, 187: 314.
